# Effect of Polydextrose on Subjective Feelings of Appetite during the Satiation and Satiety Periods: A Systematic Review and Meta-Analysis

**DOI:** 10.3390/nu8010045

**Published:** 2016-01-14

**Authors:** Alvin Ibarra, Nerys M. Astbury, Kaisa Olli, Esa Alhoniemi, Kirsti Tiihonen

**Affiliations:** 1 Active Nutrition, DuPont Nutrition & Health, Sokeritehtaantie 20, Kantvik 02460, Finland; kaisa.olli@dupont.com (K.O.); kirsti.tiihonen@dupont.com (K.T.); 2 Nuffield Department of Primary Care Health Sciences, University of Oxford, Radcliffe Observatory Quarter, Woodstock Road, Oxford OX2 6GG, UK; nerys.astbury@gmail.com; 3 Avoltus Oy, Joukahaisenkatu 1 C, Turku 20520, Finland; esa.alhoniemi@avoltus.com

**Keywords:** appetite, iAUC, meta-analysis, polydextrose, VAS

## Abstract

Introduction: Subjective feelings of appetite are measured using visual analogue scales (VAS) in controlled trials. However, the methods used to analyze VAS during the Satiation (pre- to post-meal) and Satiety (post-meal to subsequent meal) periods vary broadly, making it difficult to compare results amongst independent studies testing the same product. This review proposes a methodology to analyze VAS during both the Satiation and Satiety periods, allowing us to compare results in a meta-analysis. Methods: A methodology to express VAS results as incremental areas under the curve (iAUC) for both the Satiation and Satiety periods is proposed using polydextrose as a case study. Further, a systematic review and meta-analysis on subjective feelings of appetite was conducted following the PRISMA methodology. Meta-analyses were expressed as Standardized Mean Difference (SMD). Results: Seven studies were included in the meta-analysis. There were important differences in the methods used to analyze appetite ratings amongst these studies. The separate subjective feelings of appetite reported were Hunger, Satisfaction, Fullness, Prospective Food Consumption, and the Desire to Eat. The method proposed here allowed the results of the different studies to be homogenized. The meta-analysis showed that Desire to Eat during the Satiation period favors polydextrose for the reduction of this subjective feeling of appetite (SMD = 0.24, *I^2^* < 0.01, *p* = 0.018); this effect was also significant in the sub-analysis by sex for the male population (SMD = 0.35, *I^2^* < 0.01, *p* = 0.015). There were no other significant results. Conclusion: It is possible to compare VAS results from separate studies. The assessment of iAUC for both the Satiation and Satiety periods generates results of homogeneous magnitudes. This case study demonstrates, for the first time, that polydextrose reduces the Desire to Eat during the Satiation period. This may explain, at least in part, the observed effects of polydextrose on the reduction of levels of energy intake at subsequent meals.

## 1. Introduction

The definition of appetite covers the whole field of food intake including selection, motivation, and preference. It also refers specifically to qualitative aspects of eating, sensory aspects, and responsiveness to environmental stimulation that can be contrasted with the homeostatic view based on eating in response to physiological stimuli and an energy deficit [1]. Appetite is measured by several means including the assessment of subjective feelings, food intake, gastrointestinal hormones, and the gastric emptying rate, to mention the most relevant ones.

Satiation and Satiety are processes that govern the body’s appetite control system [2]. Satiation is the process that leads to the termination of eating, controlling the meal size [1,2]; while Satiety is the process that leads to the inhibition of further eating, a decline in hunger, and an increase in the feeling of fullness after a meal has finished [1,2]. Satiation and Satiety are both influenced by energy density, macronutrient composition, physical structure, and the sensory quality of ingested food.

Subjective feelings of appetite typically include notions of hunger, fullness, satisfaction, as well as the desire to eat, and the prospective amount of food that an individual is willing to eat [3]. Basic scale designs for capturing self-reports of subjective feelings of appetite comprise uni- and bipolar structured and unstructured lines, verbal categories, and numerical scoring [1]. The most common method is the unipolar unstructured line known as visual analogue scale (VAS), which is most often composed of lines with words anchored at each end which describe the extremes; subjects are asked to mark the line corresponding to their feelings [1,3]. Subjective feelings of appetite are typically assessed to observe the effects of foods on appetite before and after its consumption. However, on a VAS, the magnitudes of the feelings during the Satiation and Satiety periods have opposite directions, *i.e.*, during the Satiation, the maximum intensity that a food can provide is reached. During the Satiety, that intensity fades. Therefore, it is of interest to develop methodologies that allow for the measurement of the subjective feelings of appetite during each period. Further, this will facilitate the comparison of results by a means of a meta-analysis.

The methodology used to conduct a meta-analysis is well-established [4]. However, it requires that all studies are reported using the same measurement scales in order to make a fair comparison amongst them. This is challenging in the case of VAS results when different methodologies have been used in independent studies for the same food product. Moreover, not all methodologies allow for a meaningful comparison of results for the Satiation and Satiety periods. For instance, the parametric Student’s *t*-test is a classical method used to compare specific time points. Their paired or impaired variants can be used to estimate if the baselines of two individual studies are homogeneous depending on the study design, though it is often used to compare several time points independently along the trial [1]. However, analyzing appetite ratings data on a single time point basis does not take into account that appetite responses are a function of multiple time points, and these time points are not physiologically or statistically independent.

Perhaps, the most widespread method to analyze VAS (based on the number of citations found during the screening of this review) is the parametric analysis of variance (ANOVA), which allows us to compare results without further adjustment of the original data [3]. It is used to compare pre-meal values, pre- to post-prandial effects (Satiation), and post-prandial effects (Satiety). Nevertheless, it only indicates if there is a difference between groups and does not allow us to quantify any differences in magnitude (*i.e.*, % of increase or decrease of an appetite feeling with respect to a control). It has also not facilitated the comparison among independent studies with different lengths of VAS. An extension of this method is the repeated measures analysis of covariance (RMANCOVA). This mixed model approach allows us to include covariates in the analysis (e.g., baseline, age, BMI, *etc.*) [1,5]. Despite that, it is common that the pre-meal time point is used as a baseline in this method, which mixes the effects of the Satiation and Satiety periods within the analysis.

Recently, the time to return to baseline (TRTB) method has gained notoriety. It is used to estimate the time it takes to return to the initial level of appetite after food has been eaten [1,6]. However, inconveniently, the individual response curves rarely have a clear *U*-shape [1] but applying Weibull modelling gives us a meaningful statistical and practical approach [6]. Nevertheless, this method does not allow us to estimate the effects during the Satiation and Satiety periods independently, nor does it allow us to observe the entire effect of a specific food during the Satiety period.

The estimation of the area under the curve (AUC) has been extensively used in the analysis of VAS [1,7]. It has the advantage of producing a unique magnitude that integrates both the intensity of the subjective feeling of appetite and the length of the test, making it an ideal candidate for comparing independent studies of different test period lengths. However, AUC does not adjust to baseline, making it difficult to compare studies using different lengths of VAS scales (e.g., 100 mm paper and pencil *vs.* 64 mm electronic system). Further, the AUC usually reports both Satiation and Satiety as integrated periods in the analysis.

The incremental AUC (iAUC) has many characteristics of the AUC but has the advantage of adjusting the values at baseline [7]. Unfortunately, in many studies, this adjustment is done at the pre-meal timepoint, integrating both Satiation and Satiety periods in the analysis. It would be preferrable to analyze them separately. Therefore, the aim of this review is to find and propose a methodology based on iAUC during the individual Satiation and Satiety periods. This will generate homogenous magnitudes from the independent studies and allow us to compare the subjective feelings of appetite in a meta-analysis.

The use of iAUC/AUC in meta-analysis may seem challenging due to the differences in observation period length in the studies. However, since all the observation periods of the studies contain the phenomena of interest and the use of Standardized Mean Difference (SMD) in calculation of the effect size compensates for the differences in the experimental settings of the studies, commensurable effect sizes for the studies are obtained [8].

The studies conducted on appetite suppression using polydextrose are proposed as a case study. Recently, a meta-analysis showed that polydextrose effectively reduces voluntary energy intake at a subsequent meal, especially when it is administered as part of a mid-morning preload before an *ad libitum* lunch [9]. Polydextrose has also been associated with lower levels of ghrelin, and higher increases in glucagon-like peptide 1 (GLP-1) and peptide tyrosine-tyrosine (PYY) [10,11]. This evidence makes this food ingredient an ideal candidate to test new methodological concepts to assess appetite suppression.

## 2. Methodology

### 2.1. Protocol Registration

This review was conducted according to the methodology described by the Preferred Reporting Items for Systematic Reviews and Meta-Analyses: PRISMA Statement [4]. The Protocol was registered at the International Prospective Register of Systematic Reviews (PROSPERO) with number CRD42013005261 on 9 August 2013. The methodology was used to analyze the available data on the effects of polydextrose on subjective feelings of appetite and levels of energy intake. This report communicates the results on the subjective feelings of appetite. Results on different levels of energy intake are communicated in a separate report [9].

### 2.2. Eligibility Criteria and Information Sources

Eligible study designs were acute or chronic, randomized, and used placebo-controlled nutritional interventions where polydextrose was administered alone or in combination with other foods or food ingredients including supplements. Participants were males and females rated as normal, overweight, or obese, but otherwise healthy. The chosen interventions were those intended to assess the effects of polydextrose on appetite ratings and energy intake when available. Subjective feelings of appetite included, but were not limited to Hunger, Satisfaction, Fullness, Prospective Food Consumption, and the Desire to Eat.

Eligible reports included papers from scientific journals, conference abstracts, and theses reported in English-language literature before 31 July 2013, except for a full report provided by Nerys Astbury originally published as an abstract [12], and a manuscript from Kaisa Olli, later published as Olli *et al.* [11]. Searches were conducted on the following databases: BIOSIS Previews, CAB Abstracts, Foodline: Science, FSTA, Medline, SciSearch, Science Direct, Wiley Online Library, and www.ClinicalTrials.gov. Further information on recently completed trials, unpublished research, and research reported in grey literature was identified by searches for relevant documents hosted on Google Scholar.

Table S1 of the Supplementary Materials shows the generic search strategy used in the screening of studies.

### 2.3. Study Selection and Quality Assessment

One researcher (Alvin Ibarra) screened and selected the records. The authors of the selected original articles were requested to provide any missing information and full data sets on anthropometric measurements, subjective feelings of appetite, and the levels of energy intake. A second independent researcher (Kaisa Olli), checked the assessment, and any discrepancies were resolved by consulting a third researcher (Kirsti Tiihonen). The reviewed articles that were considered not relevant for this study were noted along with the reason for their exclusion.

A similar system was followed to assess the risk bias of each included study. The assessment followed the procedure described in the Cochrane Handbook for Systematic Reviews of Interventions [8].

### 2.4. Strategy for Data Synthesis

The data analysis was divided into two main sections. First, a narrative synthesis focusing on the subjective feelings of appetite was conducted to compare the methodologies used. For example, the description of the way that subjective feelings of appetite were measured was scrutinized, the kinds of foods that were provided during the studies, and the manner in which polydextrose was administered were considered. The second section focused on the analysis of the subjective feelings of appetite using the methodologies proposed in this review.

### 2.5. Definition of Satiation and Satiety Periods

The periods are defined as follows:

*Satiation*: VAS scores immediately before to immediately after the meal consumption.

*Satiety*: VAS scores immediately after the meal consumption until immediately before the next meal.

For practical reasons, all points of measurement before or after both periods (in case they exist) were discarded or treated as separate cases.

### 2.6. iAUC Estimation and Interpretation

Once the periods were defined, all the VAS scores were transformed to the same scale (0–100) and the post-meal time point was shifted to time point 0. Then, the VAS score at time point 0 was subtracted from all the VAS scores in the Satiation and Satiety periods. In other words, the curve consisting of VAS scores was shifted to 0 in timepoint 0. After that, the iAUC values were calculated separately for the Satiation (iAUC_Satiation_) and Satiety (iAUC_Satiety_) periods using the trapezoidal rule. iAUC above the zero reference was assigned a positive value and below was assigned negative one, and both were expressed as minutes times millimeter (min.mm).

Figure S1 of the Supplementary Materials provides an example for Hunger, Prospective Food Consumption, and the Desire to Eat; Figure S2 provides an example for Satisfaction and Fullness. These figures exemplify the behavior of an ideal appetite suppressive *verum* as compared to a *placebo* control. Notice that the magnitudes for the same subjective feeling of appetite during the Satiation and Satiety periods are opposites. This is an important point to take into consideration for the correct interpretation of iAUC_Satiation_ and iAUC_Satiety_ values of a specific subjective feeling over the appetite suppressive effect of a given food product.

Using the following rule, it is possible to correct the opposite values of iAUC_Satiation_ and iAUC_Satiety_ for each subjective feeling of appetite in order to ensure that the potential effect of a food product as an appetite suppressing agent is interpreted correctly:

*Multiply iAUC_Satiation_ by −1*: to indicate that negative values for Hunger “reduce hunger”; for Prospective Food Consumption, “lower the amount expected to eat”; and for the Desire to Eat, “reduce the desire to eat”.

*Multiply iAUC_Satiety_ by −1*: to indicate that positive values for Satisfaction “increase satisfaction”; and for Fullness, “increase fullness”.

Note that the adjustments made to the methodology do not affect the significance of the meta-analysis.

### 2.7. Meta-Analysis

For this meta-analysis, data sets were investigated using a random effects model which considers our chosen studies to be a sample of a larger universe of studies. The model was chosen because there were minor differences in both study design and the participants’ characteristics. Therefore, a common effect size could not be assumed for all the studies. The treatment-effect size was analyzed using SMD with a 95% confidence interval. The effect size was estimated using Hedges’ g measure. The between-study variation was estimated using a restricted maximum likelihood approach. The results of the meta-analysis was visualized using a Forest plot, which illustrates the results of the individual studies as well as the summary random effect. The numbers of effect sizes, *K*, were reported; and the total heterogeneity in the dataset was tested using the *Q* and Higgins *I^2^* statistics. The publication bias was analyzed visually using Funnel plots and assessed using the Egger’s test.

All statistical analyses were performed using software “R” version 3.2.2 (R Core Team, R Foundation for Statistical Computing, Vienna, Austria) [13], and metafor package version 1.9-8 (Wolfgang Viechtbauer, Maastricht, The Netherlands) [14]. The “R” code is available online at https://github.com/avoltusfi/pdx_vas_meta.

## 3. Results

### 3.1. Included Studies

Table S2 of the Supplementary Materials shows the number of hits obtained in each database during the screening. Twenty-two complete studies on the effects of polydextrose on energy intakes and subjective feelings of appetite were assessed for eligibility for inclusion in the systematic review and meta-analysis using the same criteria. The meta-analysis on energy intakes is published [9]. In the present report on subjective feelings of appetite, fifteen studies were excluded with reasons as provided by Ibarra *et al.* [9], the lack of original data being the main cause for exclusion. Most of the data used in this meta-analysis was delivered by authors of the original publications under confidentiality agreements.

**Figure 1:**
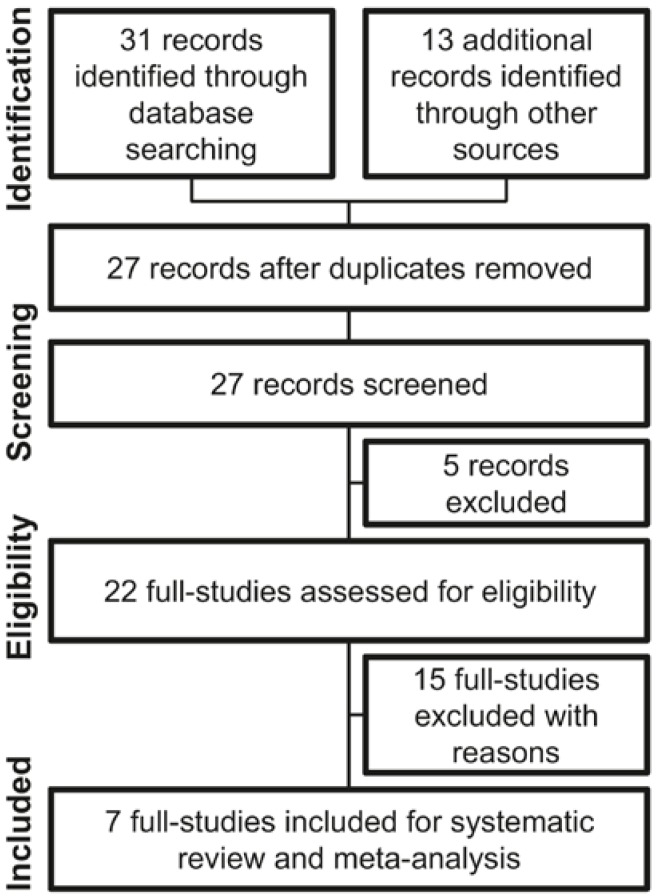
Preferred Reporting Items for Systematic Reviews and Meta-Analyses (PRISMA) flow information diagram used to select studies on the effects of polydextrose on subjective feelings of appetite for this review.

The flow diagram on Figure 1 demonstrates the screening process used to determine how the included studies were selected.

Seven studies were included for the assessment of subjective feelings of appetite: Olli *et al.* (2015) [11], Astbury *et al.* (2013) [15], Ranawana *et al.* (2013) [16], Hull *et al.* (2012) [17], Astbury *et al.* (2008) [12], Schwab *et al.* (2006) [18] and King *et al.* (2005) [19]. In the previous meta-analysis on energy intakes [9], two of these studies were not included [11,18] because they did not measure food intake. The original data on subjective feelings of appetite of one of the studies [10] was not accesible.

The studies included in this analysis represent a universe of 135 participants, of which 66 were males and 59 were females. The doses of polydextrose tested in these studies ranged from 6.25 to 25.0 grams. Table 1 summarizes the design, procedures, and main outcomes of these studies with respect to subjective feelings of appetite.

### 3.2. Risk of Bias

The only double-blind studies included in this analysis were conducted by Olli *et al.* [11] and Schwab *et al.* [18]. All other studies were single-blinded, meaning that whilst the volunteers were unaware of the treatments they were given, the investigators were aware. Single-blinded studies are susceptible to a high risk of bias. Further, King *et al.* [19] communicated that sixteen volunteers were enrolled in the study; but a later revision of the clinical report revealed that results on subjective feelings of appetite were calculated using only fourteen participants (7 male and 7 female), and results on energy intake on fifteen participants (Table 1**)**. The reasons for these discrepancies were not clarified in the original report leading to a high risk of bias for this study.

All studies included in this review used commercial polydextrose, Litesse Ultra^®^, or Litesse Two^®^, manufactured by DuPont. Studies conducted by Astbury *et al.* [12,15], were sponsored by the Biotechnology and Biological Sciences Research Council (BBSRC) of the United Kingdom and Mars UK acting as a private partner. All other studies were sponsored, at least in part, by DuPont.

### 3.3. Qualitative Results of Appetite Ratings

Five subjective feelings of appetite were identified and assessed using visual analogue scales (VAS). All studies measured Hunger, six studies looked at Fullness and the Desire to Eat, three studies evaluated Satisfaction, and two studies examined Prospective Food Consumption. Table 2 summarizes the methodology used to measure each of these subjective feelings of appetite. Some studies reported other parameters as measured by VAS, which were not necessarily related to appetite, such as assessment of participants’ well-being or sensory characteristics of investigational products. These extraneous parameters were, therefore, excluded from this review.

**Table 1:** Summary of studies included for systematic review and meta-analysis of subjective feelings of appetite.

Study	Investigational Products	Population	Design	Procedure	Main Outcomes on Subjective Feelings of Appetite
Olli *et al.* [11]	400 mL cola drink (0.0 g PDX) 400 mL cola drink (15.0 g PDX) Cola drinks (833 kJ and 800 kJ, with and without PDX, Litesse Ultra^®^, DuPont) were consumed together with a hamburger (2071 kJ) and French fries (1423 kJ)	5 Men (41.4 years, 33.2 Kg/m^2^) 13 Women (42.7 years, 33.8 Kg/m^2^) 18 Total (42.0 years, 33.6 Kg/m^2^)	Acute, randomized, double-blinded, placebo-controlled, and crossover (10 days of washout) study conducted in Kuopio and Vierumäki, FI	Participants were advised to avoid strenuous exercise and not to drink alcohol 24 h before the test day. Experiment began the next morning after a 10–12 h fast. An intravenous catheter was inserted in the antecubital vein and then participants were asked to eat the meal in 20 min. Blood samples were taken five times after the study meal (at 30, 60, 120, 240, and 360 min). Appetite ratings were measured before the meal and 40, 70, 140, and 280 min after. Post-meal energy intakes were not measured in this study.	PDX reduced iAUC for Hunger by 40% (*p* = 0.03) and marginally increased Satisfaction by 22.5% (*p* = 0.08) during the post-meal satiety period.
Astbury *et al.* [15]	400 mL preload (0.0 g PDX) 400 mL preload (6.3 g PDX) 400 mL preload (12.5 g PDX) 400 mL preload (25.0 g PDX) Preloads were chocolate-flavored liquids (837 kJ). PDX (Litesse Ultra^®^, DuPont) was compensated with maltodextrin in the control.	12 Men (22.5 years, 23.2 Kg/m^2^) 9 Women (24.7 years, 22.3 Kg/m^2^) 21 Total (23.3 years, 22.3 Kg/m^2^)	Acute, randomized, single-blinded, placebo-controlled, and crossover (1 week of washout) study conducted in Nottingham, UK.	Participants were asked to consume a standardized dinner at 20:00 the day before the test and to refrain from consuming alcohol and undertaking vigorous exercise. The day of the test, they had a standardized breakfast at home (10% daily energy expenditure), arrived at the laboratory at 10:45 and were served a preload at 11:00. Appetite ratings were collected at baseline, 0 min (immediately after preload) and 30, 60, 90 min later, and 0 (immediately after), 30 and 60 min after the test meal. The test meal was *ad libitum* (657 kJ/100 g). Then, participants were instructed to complete food diaries for the rest of the day.	There were no significant differences on subjective feelings of appetite between PDX groups and control.
Ranawana *et al.* [16]	400 g preload (0.0 g PDX) 400 g preload (12.0 g PDX) Preloads were fruit smoothies (870.8 kJ and 883.4 kJ for the control and treatment, respectively). PDX (Litesse Two^®^, DuPont) was not compensated in the control.	26 Men (28.0 years, 24.1 Kg/m^2^) 0 Women 26 Total (28.0 years, 24.1 Kg/m^2^)	Acute, randomized, single-blinded, placebo-controlled, and crossover (>2 days of washout) study conducted in Oxford, UK.	Participants were asked to refrain from consuming alcohol and undertaking vigorous exercise. The day of the test, they arrived at 8:00 at the laboratory, after 10 h fast. Appetite ratings were determined before the breakfast, after the breakfast, 1 h after the breakfast, before preload and 15, 30, 45 and 60 (just before *ad libitum* lunch) min after the preload, and after the lunch. The size of the first breakfast meal was measured and the same size was used for the next session.	There were no significant differences on subjective feelings of appetite between the PDX group and control.
Hull *et al.* [17]	200 g preload (0.0 g PDX) 200 g preload (6.25 g PDX) 200 g preload (12.5 g PDX) Preloads were yogurts (671 kJ). PDX (Litesse Two^®^, DuPont) was compensated with glucose syrup in the control.	10 Men (32.8 years, 23.8 Kg/m^2^) 24 Women (38.7 years, 22.5 Kg/m^2^) 34 Total (36.9 years, 22.9 Kg/m^2^)	Acute, randomized, single-blinded, placebo-controlled, and crossover (1 week of washout) study conducted in Surrey, UK.	Participants were asked to consume a standardized dinner at 20:00 the day before the test and to refrain from consuming alcohol and undertaking vigorous exercise. The day of the test, they arrived at the laboratory at 08:00 and were served a breakfast (consistent meal sizes were used for subsequent sessions). Appetite ratings were collected at 0 (before breakfast), 15 (after breakfast), 45, 75, 105, 135, 150 (before preload), 165 (after preload), 180, 195, 210, 225, 240 (before lunch), 270 (after lunch), 300, 330, 360, 390, 420, 450, 480, 510, 540, 570 (before dinner), and 600 (after dinner) min. Lunch (984 kJ/100 g) and dinner (523 kJ/100 g) were *ad libitum*.	In the period between preload and lunch, 6.25 g PDX reduced the Desire to Eat (*p* < 0.001), Hunger (*p* < 0.03), and increased Satisfaction (*p* < 0.02), while 12.5 g PDX also reduced Hunger (*p* < 0.02) and Prospective Food Consumption (*p* < 0.03). In the period between preload and dinner 6.25 g PDX reduced the Desire to Eat (*p* = 0.002), and 12.5 g PDX increased Satisfaction (*p* < 0.006); however, both concentrations reduced Fullness (*p* < 0.05).
Astbury *et al.* [12]	400 mL preload (0.0 g PDX) 400 mL preload (25.0 g PDX) Preloads were chocolate-flavored milk shake (1047 kJ). PDX (Litesse Ultra^®^, DuPont) was compensated with maltodextrin in the control.	14 Men (25.3 years, 23.0 Kg/m^2^) 0 Women 14 Total (25.3 years, 23.0 Kg/m^2^)	Acute, randomized, single-blinded, placebo-controlled, and crossover (1 week of washout) study conducted in Nottingham, UK.	Participants were asked to consume a standardized dinner at 20:00 the day before the test and to refrain from consuming alcohol and undertaking vigorous exercise. The day of the test, they had a standardized breakfast at home (10% daily energy expenditure), arrived to the laboratory at 10:45 and were served a preload at 11:00. Appetite ratings were collected at baseline, 0 min (immediately after preload) and 30, 60, 90 min later, and 0 (immediately after), 30 and 60 min after the test meal. The test meal was *ad libitum* (657 kJ/100 g). Then, participants were instructed to complete food diaries for the rest of the day.	There were no significant differences on subjective feelings of appetite between the PDX group and control.
Schwab *et al.* [18]	400 mL drink (0.0 g PDX or SPB) 400 mL drink (16.0 g PDX) 400 mL drink (16.0 g SBP) Drinks contained 469 kJ, 603 kJ and 610 kJ for the control, PDX (Litesse Ultra^®^, DuPont) and SBP, respectively	6 Men (55.3 years, 30.0Kg/m^2^); 18 Women (53.8 years, 29.0Kg/m^2^) 24 Total (54.2 years, 29.2 Kg/m^2^) 8 participants were assigned to each group—This is a subgroup of a 66 participants study	Chronic (12 weeks), randomized, double-blinded and parallel study conducted in Kuopio, FI	Appetite ratings were measured in the subgroup at the beginning (week 0) and at the end (week 12) of the clinical intervention. At week 0, participants consumed only half of the daily dose (200 mL). Participants arrived to the lab after 12 h overnight fasting. Appetite ratings were measured before and after 15, 30, 60, 120, and 180 min of the standardized breakfast. Post-meal energy intakes were not measured in this study.	No differences were found on appetite ratings in the subgroup except for feeling of Hunger (*p* < 0.05) at 180 min within the PDX group (6.0 ± 3.6 *vs*. 3.3 ± 2.5, week 0 *vs*. week 12, respectively).
King *et al.* [19]	200 g preload (0.0 g PDX or XYL) 200 g preload (25.0 g XYL) 200 g preload (25.0 g PDX) 200 g preload (12.5 g PDX + 12.5 g XYL) Preloads were yogurts containing 854 kJ, 686 kJ, 544 kJ, and 611 kJ for the control, XYL, PDX, and the combination, respectively. In the control, PDX (Litesse Ultra^®^, DuPont) was compensated with sucrose.	7 Men (30.7 years, 23.8 Kg/m^2^) 8 Women (29.5 years, 21.6 Kg/m^2^) 15 Total (30.1 years, 22.7 Kg/m^2^)	Chronic (10 days), randomized, single-blinded, placebo controlled and crossover study conducted in Leeds, UK.	On days 1 and 10, participants had a breakfast at 8:30 at the laboratory (consistent meal sizes were used for subsequent sessions). They were instructed to consume the preload at 11:00 and not to consume any other food or drink between the breakfast and lunch interval. Appetite ratings were measured at 0 (before breakfast), 15 (after breakfast), 90, 150 (before preload), 165 (after preload), 210, 240 (before *ad libitum* lunch), 255 (after *ad libitum* lunch), 330, 390, 450, 540 (before diner), 555 (after diner), and 810 min. On days 2–9 participants were requested to drink a preload at 11:00 daily and to complete test food intake questionnaires.	There was no significant difference on subjective feelings of appetite between the PDX group (25.0 g) and control.

iAUC = Incremental Area Under the Curve; FI = Finland; PDX = Polydextrose; SBP = Sugar Beet Pectin; UK = United Kingdom; VAS = Visual Analogue Scale; XYL = Xylitol.

**Table 2:** Characteristics of the methodologies to measure subjective feelings of appetite used in the studies selected for this review.

Study	Period	Method	Question	Lower Set	Upper Set	Scale Length/Magnitude	System	Statistical Analysis
**Hunger**								
Olli *et al.* [11]	40 min (Satiation); 280 min (Satiety)	[3,20]	Kuinka nälkäiseksi tunnet itsesi tällä hetkellä? *How hungry do you feel at the moment?*	En ole lainkaan nälkäinen *I am not at all hungry*	Olen erittäin nälkäinen *I am very hungry*	100 mm	Paper and pencil	iAUC for the Satiation and Satiety periods and compared groups using Student’s paired *t*-test
Astbury *et al.* [12,15]	~15 min (Satiation); 90 min (Satiety); 165 min (full experiment)	[21,22]	How hungry do you feel?	Not at all	Extremely	100 mm/500 points	Electronic-Suss-ex Ingestion Pattern Monitor	Changes from baseline (between preload to the next meal) using two-way repeated measures ANOVA
Ranawana *et al.* [16]	~15 min (Satiation); 60 min (Satiety)	[2,7]	How hungry do you feel?	Not at all hungry	Extremely hungry	100 mm	Paper and pencil	iAUC and compared groups using Student’s paired *t*-test
Hull *et al.* [17]	~15 min (Satiation); 90 min (Satiety); 450 min (full experiment)	[3]	How hungry are you?	Not at all	Extremely	64 mm/100 points	Electronic-hand held computer iPAQs	Changes from baseline (between preload to the next meal) using two-way repeated measures ANOVA
Schwab *et al.* [18]	~15 min (Satiation); 180 min (Satiety)	[3]	Kuinka nälkäiseksi tunnet itsesi? *How hungry do you feel?*	En ole lainkaan nälkäinen *I am not hungry at all*	Olen niin nälkäinen kuin voin olla *I am as hungry as I can be*	100 mm	Paper and pencil	Specific time points comparison between groups using Student’s paired *t*-test
King *et al.* [19]	~15 min (Satiation); 90 min (Satiety); 810 min (full experiment)	[23,24]	How hungry do you feel?	Not at all hungry	As hungry as I've ever felt	66 mm/100 points	Electronic Appetite Ratings System (EARS)	Changes from baseline (between preload to the next meal) using two-way repeated measures ANOVA
**Satisfaction**								
Olli *et al.* [11]	40 min (Satiation); 280 min (Satiety)	[3,20]	Kuinka kylläiseksi tunnet itsesi tällä hetkellä? *How satisfied do you feel at the moment?*	En ole lainkaan kylläinen *I do not feel satisfied at all*	Olen erittäin kylläinen *I feel very satisfied*	100 mm	Paper and pencil	iAUC for the Satiation and Satiety periods and compared groups using paired Student’s *t*-test
Hull *et al.* [17]	~15 min (Satiation); 90 min (Satiety); 450 min (full experiment)	[3]	How satiated are you?	Not at all	Extremely	64 mm/100 points	Electronic-hand held computer iPAQs	Changes from baseline (between preload to the next meal) using two-way repeated measures ANOVA
Schwab *et al.* [18]	~15 min (Satiation); 180 min (Satiety)	[3]	Kuinka kylläiseltä olosi tuntuu? *How satisfied do you feel?*	En ole lainkaan kylläinen *I am not satisfied at all*	Olen niin kylläinen kuin voin olla *I am as satisfied as I can be*	100 mm	Paper and pencil	Specific time points comparison between groups using Student’s paired *t*-test
**Fullness**								
Astbury *et al.* [12,15]	~15 min (Satiation); 90 min (Satiety); 165 min (full experiment)	[21,22]	How full do you feel?	Not at all	Extremely	100 mm/500 points	Electronic-Suss-ex Ingestion Pattern Monitor	Changes from baseline (between preload to the next meal) using two-way repeated measures ANOVA
Ranawana *et al.* [16]	~15 min (Satiation); 60 min (Satiety)	[2,7]	How full do you feel?	Not at all full	Extremely full	100 mm	Paper and pencil	iAUC and compared groups using Student’s paired *t*-test
Hull *et al.* [17]	~15 min (Satiation); 90 min (Satiety); 450 min (full experiment)	[3]	How full are you?	Not at all	Extremely	64 mm/100 points	Electronic-hand held computer iPAQs	Changes from baseline (between preload to the next meal) using two-way repeated measures ANOVA
Schwab *et al.* [18]	~15 min (Satiation); 180 min (Satiety)	[3]	Kuinka täydeltä olosi tuntuu? *How full do you feel?*	Ei lainkaan täydeltä *Not full at all*	Niin täydeltä kuin vain voi tuntua *As full as one can be*	100 mm	Paper and pencil	Specific time points comparison between groups using Student’s paired *t*-test
King *et al.* [19]	~15 min (Satiation); 90 min (Satiety); 810 min (full experiment)	[23,24]	How full do you feel?	Not at all full	As full as I've ever felt	66 mm/100 points	Electronic Appetite Ratings System (EARS)	Changes from baseline (between preload to the next meal) using two-way repeated measures ANOVA
**Prospective Food Consumption**								
Ranawana *et al.* [16]	~15 min (Satiation); 60 min (Satiety)	[2,7]	How much food do you think you can eat?	Nothing at all	A large amount	100 mm	Paper and pencil	iAUC and compared groups using Student’s paired *t*-test
Hull *et al.* [17]	~15 min (Satiation); 90 min (Satiety); 450 min (full experiment)	[3]	How much do you think you could eat right now?	Nothing at all	A very large amount	64 mm/100 points	Electronic-hand held computer iPAQs	Changes from baseline (between preload to the next meal) using two-way ANOVA
**Desire to Eat**								
Olli *et al.* [11]	40 min (Satiation); 280 min (Satiety)	[3,20]	Kuinka voimakas on halusi syödä tällä hetkellä? *How strong is your desire to eat at the moment?*	Minulla ei ole lainkaan halua syödä *I do not have desire to eat at all*	Haluni syödä on erittäin voimakas *My desire to eat is very strong*	100 mm	Paper and pencil	iAUC for the Satiation and Satiety periods and compared groups using Student’s paired *t*-test
Astbury *et al.* [12,15]	~15 min (Satiation); 90 min (Satiety); 165 min (full experiment)	[21,22]	How much of a desire to eat do you feel?	Not at all	Extremely	100 mm/500 points	Electronic-Suss-ex Ingestion Pattern Monitor	Changes from baseline (between preload to the next meal) using two-way repeated measures ANOVA
Ranawana *et al.* [16]	~15 min (Satiation); 60 min (Satiety)	[2,7]	How strong is your desire to eat?	Not at all strong	Extremely strong	100 mm	Paper and pencil	iAUC and compared groups using Student’s paired *t*-test
Hull *et al.* [17]	~15 min (Satiation); 90 min (Satiety); 450 min (full experiment)	[3]	How strong is your desire to eat?	Very weak	Very strong	64 mm/100 points	Electronic-hand held computer iPAQs	Changes from baseline (between preload to the next meal) using two-way repeated measures ANOVA
Schwab *et al.* [18]	~15 min (Satiation); 180 min (Satiety)	[3]	Kuinka suuri on halusi syödä juuri nyt? *How great is your desire to eat right now?*	En haluaisi syödä mitään *I would not want to eat anything*	Haluni syödä on niin suuri kuin vain voi olla *My desire to eat is as great as it can be*	100 mm	Paper and pencil	Specific time points comparison between groups using Student’s paired *t*-test

ANOVA = Analysis of Variance; IAUC = Incremental Area under the Curve; Satiation = period between immediately before and immediately after treatment intake; Satiety = period between treatment in take and the subsequent meal.

### 3.4. iAUCSatiation and iAUCSatiety

The iAUC_Satiation_ and iAUC_Satiety_ values for the polydextrose and the control groups are reported in the Supplementary Materials for Hunger (Table S3), Satisfaction (Table S4), Fullness (Table S5), Prospective Food Consumption (Table S6), and the Desire to Eat (Table S7).

### 3.5. Meta-Analyses of Subjective Feelings of Appetite

Four indicators for the subjective feelings of appetite were included in the meta-analysis: Hunger, Satisfaction, Fullness, and the Desire to Eat. There were two studies that assessed Prospective Food Consumption (Hull *et al.* [17] and Ranawana *et al.* [16]), but their reporting of results was not consistent; therefore, they could not be analyzed together.

**Figure 2:**
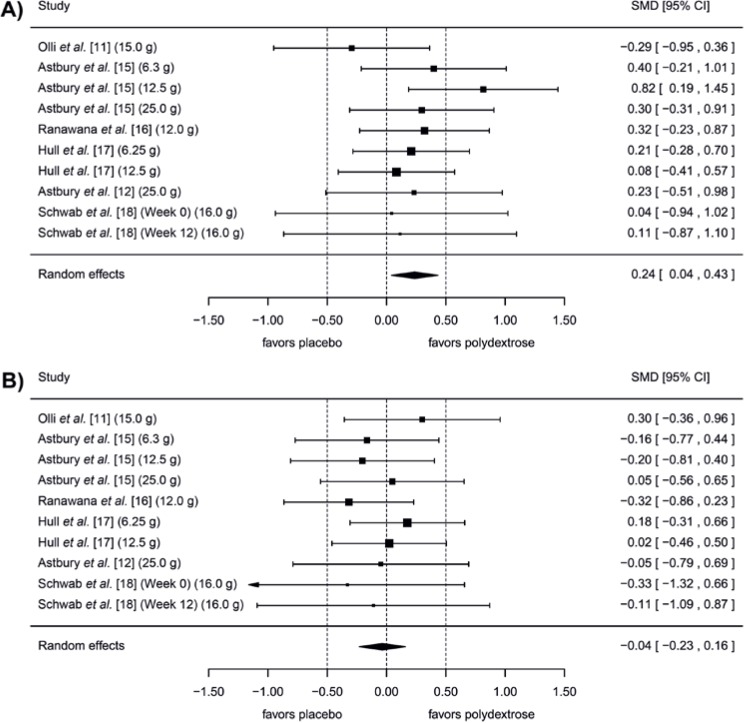
Meta-analysis comparing doses of polydextrose *versus* placebo in the studies selected for this review on the subjective feelings of Desire to Eat, adjusted to show “less desire to eat with polydextrose” if the Standardized Mean Difference calculated using Hedges’ g measure (SMD, 95% CI) favors it, (**A**) during the Satiation period and (**B**) during the Satiety period. Doses of polydextrose per day used in each treatment are presented in brackets next to each reference.

Results of the meta-analysis are summarized in Table 3. The only significant result (*p* = 0.018) in the whole group was observed with the Desire to Eat category with small effect size (0.24) [25]. Here, the results of the random effects model indicate that the meta-analysis significantly favors polydextrose for the “reduction of the desire to eat” over the placebo during the Satiation period (Figure 2). The Higgins *I^2^* statistic for this variable was zero, evidencing the high consistency of the data. In addition, the indicator of Egger’s test was not significant, confirming a low level of bias. The results of the other indicators of subjective feelings of appetite, including the Funnel plots, are presented in the Supplementary Materials (Figures S3–S9).

**Table 3:** Meta-analysis, by sex, of subjective feelings of appetite for the Satiation and Satiety periods of the polydextrose studies selected for this review.

Groups	*n*	SMD (95% CI)	*p*-Value	*I^2^*	Egger’s Test
**Whole group**					
*Satiation*					
Hunger	135	−0.05 (−0.23, 0.14)	0.60	< 0.01	0.58
Satisfaction	60	−0.08 (−0.37, 0.20)	0.56	<0.01	0.35
Fullness	117	0.05 (−0.14, 0.24)	0.61	<0.01	0.021 (*)
Desire to Eat	121	0.24 (0.04, 0.43)	0.018 (*)	<0.01	0.81
*Satiety*					
Hunger	135	0.11 (−0.07, 0.29)	0.24	<0.01	0.040 (*)
Satisfaction	60	0.16 (−0.11, 0.44)	0.25	<0.01	0.33
Fullness	117	−0.03 (−0.23, 0.16)	0.72	<0.01	0.10
Desire to Eat	121	−0.04 (−0.23, 0.16)	0.72	<0.01	0.42
**Males**					
*Satiation*					
Hunger	76	0.07 (−0.19, 0.33)	0.59	<0.01	0.26
Satisfaction	17	−0.12 (−0.68, 0.45)	0.69	<0.01	0.22
Fullness	71	0.08 (−0.19, 0.34)	0.58	<0.01	0.69
Desire to Eat	69	0.35 (0.07, 0.63)	0.015 (*)	<0.01	0.42
*Satiety*					
Hunger	76	0.07 (−0.18, 0.33)	0.57	<0.01	0.10
Satisfaction	17	0.36 (−0.18, 0.90)	0.19	<0.01	0.38
Fullness	71	0.01 (−0.25, 0.28)	0.94	<0.01	0.94
Desire to Eat	69	−0.02 (−0.30, 0.25)	0.87	<0.01	0.023 (*)
**Females**					
*Satiation*					
Hunger	59	−0.15 (−0.42,0.11)	0.25	<0.01	0.24
Satisfaction	43	−0.09 (−0.42, 0.24)	0.59	<0.01	0.33
Fullness	46	0.00 (−0.27, 0.28)	0.99	<0.01	0.014 (*)
Desire to Eat	52	0.15 (−0.13, 0.43)	0.29	<0.01	0.84
*Satiety*					
Hunger	59	0.14 (−0.12, 0.41)	0.28	<0.01	0.030 (*)
Satisfaction	43	0.13 (−0.20, 0.45)	0.45	<0.01	0.65
Fullness	46	−0.08 (−0.39, 0.23)	0.62	0.07	0.08
Desire to Eat	52	−0.04 (−0.32, 0.23)	0.76	<0.01	0.06

*n* = Number of participants in the included studies; SMD (95% CI) = Standardized Mean Difference at 95% Confidence Interval; *I^2^* = Higgins statistic; (*) = statistically significant, *p*-value < 0.05.

When analyzing results by sex for the category Desire to Eat, the meta-analysis favors polydextrose but only in the male population. Table 3 shows that there were no other effects on subjective feelings of appetite in the whole groups or in the sex-specific sub-groups.

## 4. Discussion

Dietary fibers such as polydextrose are thought to impact Satiation and Satiety, which are processes that are involved in the body’s appetite control system [26]. The classical definition of Satiation indicates that it leads to the termination of eating, accompanied by the satisfaction of appetite. Satiety is explained as the feeling of fullness, which hinders hunger and further consumption of food. Satiation and Satiety are both involved in limiting energy intake and are important in determining the total energy intake [2].

All the studies included in this analysis used methodologies which assess subjective feelings of appetite as validated in previous research [2,3,7,20,21,22,23,24]. Although there were some minor variations, in general, the questions for each indicator for the feeling of appetite can be considered similar. This also applies to the two studies that used inventories in Finnish language (Olli *et al.* [11] and Schwab *et al.* [18]). However, the wordings used for questions at the upper and lower ends of the response scales were less consistent among studies. The biggest variation was found between the methodologies used to record changes on subjective feelings of appetite and the ways in which results were analyzed. Three studies asked participants to use a pencil to mark their subjective feelings of appetite on a 100 mm paper scale (Olli *et al.* [11], Ranawana *et al.* [16], and Schwab *et al.* [18]), while the other four studies used electronic systems with differing scale lengths (Astbury *et al.* [12], Astbury *et al.* [15], Hull *et al.* [17], and King *et al.* [19]). Olli *et al.* [11] was the only study which reported results as iAUC for the Satiation and Satiety periods separately, while Ranawana *et al.* [16] reported results as iAUC for the full assessment of appetite feelings. Both studies compared groups using paired Student’s *t*-test. Schwab *et al.* [18] directly compared specific time points also using Student’s paired *t*-test. King *et al.* [19] compared changes from the pre- to post-load using two-way repeated measures ANOVA. All other studies analyzed changes from the subjective appetite responses baseline to the preload (between test product to the next meal) using two-way repeated measures ANOVA (Astbury *et al.* [12], Astbury *et al.* [15] and Hull *et al.* [17]).

Two included studies measured subjective feelings of appetite acutely before and after a chronic administration of polydextrose: King *et al.* [19] in a crossover design and Schwab *et al.* [18] in a parallel design. In these interventions the same volunteers participated in the pre- and post-measurements. The results on appetite ratings between these two time points varied to a large extent, most likely due to the effect of the chronic administration of polydextrose. In addition, the number of participants in these two studies is small and the variance is large. Hence, the total impact of the two repeated studies on the total effect was very small. Therefore, in the current report, the same criteria was followed as per the meta-analysis on different levels of energy intakes [9], where we considered each acute measurement as an independent study.

Hunger was the only indicator of subjective feelings of appetite that was assessed in all the studies that were included. The meta-analysis of the Satiation period clearly did not favor polydextrose for reducing this parameter. The meta-analysis of the Satiety period also did not show a statistical difference. However, some of the included studies did report significant changes for Hunger during the Satiety period when different analysis methodologies were used (Hull *et al.* [17] and Olli *et al.* [11]). Future studies may help to confirm the tendency of polydextrose to reduce Hunger during the Satiety period.

Only three studies measured Satisfaction (Hull *et al.* [17], Olli *et al.* [11] and Schwab *et al.* [18]). The meta-analysis did not favor polydextrose during the Satiation or the Satiety periods for the increase of this parameter. However, two of the included studies reported that polydextrose increased Satisfaction significantly (Hull *et al.* [17]), or marginally (Olli *et al.* [11]), during the Satiety period. It is important to mention that the wording used to evaluate Satisfaction varies amongst the reported methodologies making it difficult to properly assess this subjective feeling of appetite. For example, some authors recommend using the term Satiety instead of Satisfaction (e.g., “How Satiated are you?” instead of “How Satisfied are you?”) [3]. Nevertheless, future studies may help to confirm the tendency of polydextrose to increase the feeling of Satisfaction during the Satiety period.

Surprisingly, the meta-analysis did not favor polydextrose or the placebo for an increase of Fullness during the Satiation or Satiety periods. Polydextrose is a well-recognized dietary fiber [27]. In general, dietary fibers are well known to increase the feeling of Fullness due to their properties of adding bulk and producing viscosity in foods [26]. However, polydextrose in the study dose range of 6.25–25.0 g/day is highly soluble and has little impact on the viscosity of beverages [28]. This may explain, at least in part, why polydextrose did not show any effect on Fullness in the meta-analysis. The only individual study that reported a reduction in Fullness was Hull *et al.* [17], but only after the *ad libitum* meal during the post-Satiety period.

Although two studies assessed Prospective Food Consumption (Hull *et al.* [17] and Ranawana *et al.* [16]), it was not possible to conduct a meta-analysis because Ranawana *et al.* [16] reported results in an non-aligned way (e.g., values for both polydextrose and control decay along the Satiety period). Hull *et al.* [17] reported that polydextrose reduced Prospective Food Consumption as compared to the placebo in the Satiety period. More studies measuring this parameter are needed in order to confirm this observation.

The most striking result was that the meta-analysis favors polydextrose for the reduction of the Desire to Eat during the Satiation period. There were no statistical differences during the Satiety period for this parameter. Six studies measured the Desire to Eat. However, only Hull *et al.* [17] reported a reduction of the Desire to Eat after the intake of polydextrose, both between the preload challenge until the subsequent meal at lunch time and after the lunch until dinner.

It has been proposed that the category of sex may play a role on the subjective feelings of appetite during the Satiety period, where generally females felt more satisfied than males [29]. However, little is known about this influence during the Satiation period. In this review, the meta-analysis shows that results by sex are comparable to the results of the entire mixed group during both the Satiation and Satiety periods, except for the Desire to Eat category during the Satiation period. Here the meta-analysis favors polydextrose only in the male population. The Desire to Eat category was assessed in six studies involving 69 males and 52 females, which represents an important part of the total universe of this review. To our knowledge, this is the first report on the effects of polydextrose on the Desire to Eat by sex. Future studies designed to assess differences on subjective feelings of appetite by sex (e.g., including equal sample sizes of males *versus* females) may help us to better understand this observation.

Results of this review indicate that some studies demonstrate that polydextrose affects several subjective feelings of appetite at doses which are in the commercial application range for foods and dietary supplements, *i.e.*, between 6.25 g and 25.0 g in a single dose per day. When high doses of polydextrose have been tested, such as 56.7 g over the duration of a day [30], its effects on subjective feelings of appetite are conclusive: it reduces Hunger and the Desire to Eat, while increasing the feeling of Fullness and Satiety (Satisfaction).

In this review, the incremental area under the curve for the Satiation (iAUC_Satiation_) and Satiety (iAUC_Satiety_) periods of subjective feelings of appetite were analyzed to investigate the appetite-suppressing effect of polydextrose. Although other meta-analyses on VAS have been conducted using iAUC [31], to our knowledge, this is the first review that investigates this effect in both periods independently. The duration of the Satiation periods were almost the same in all studies, approximately 15 min, except in Olli *et al.* [11] where the period was 40 min. The lengths during the Satiety period varied more, between 60 (Ranawana *et al.* [16]) and 280 min (Olli *et al.* [11]) among studies. The differences in duration of these periods are perhaps the biggest limitation to the methodology used in this review. The lack of consistency among participants in the same study can also be considered a problem when validating this methodology. Once all individual iAUCs are evaluated in order to generate an average value, the effects of each period are clearly visualized in both iAUC_Satiation_ and iAUC_Satiety_. The study by Olli *et al.* [11] is a good example of the methodology used for this purpose. However, inconsistent results amongst the participants may cause large deviations in the iAUC values and may produce negative values, which in turn may cause abnormal relative changes. The study by Schwab *et al.* [18] is a clear example of where this has occurred, and the methodology cannot easily be applied (see iAUC values in the Supplementary Materials). Nevertheless, inconsistent results are the exception with most of the studies, and overall, the methodology is very reliable. The estimation of iAUC allowed for the comparison of several studies using the same magnitude in this meta-analysis. Future studies may further estimate the reproducibility and validity of this methodology. Further, due to the fact that many of the included studies assessed energy intakes, the estimation of iAUC in the Satiation and Satiety periods could be complemented with the estimation of satiety quotients [32], with the aim of extending our understanding of the effects of a consumed food on appetite.

## 5. Conclusions

The methodology used to homogenize results of iAUC on the subjective feelings of appetite during the Satiation and Satiety periods allowed for a meaningful comparison of the results of the studies included in this meta-analysis. This review demonstrates, for the first time, that polydextrose reduces the Desire to Eat during the Satiation period. This effect was also significant in the sub-analysis by sex for the male population. The results on this subjective feeling of appetite may explain, at least in part, the observed effects of polydextrose on the reduction of energy intake at a subsequent meal. There were no differences on Hunger, Satisfaction, or Fullness during the Satiation or Satiety periods. A future meta-analysis incorporating more studies may confirm the tendency of polydextrose to reduce Hunger and increase Satisfaction during the Satiety period. This meta-analysis shows that polydextrose does not affect Fullness, possibly due to its high solubility and low viscosity.

## Supplementary Materials

This section is available online at http://www.mdpi.com/2072-6643/8/1/45/s1.

## References

[1] Blundell J., de Graaf C., Hulshof T., Jebb S., Livingstone B., Lluch A., Mela D., Salah S., Schuring E., van der Knaap H. (2010). Appetite control: Methodological aspects of the evaluation of foods. Obes. Rev..

[2] Benelam B. (2009). Satiation, satiety and their effects on eating behaviour. Nutr. Bull..

[3] Flint A., Raben A., Blundell J.E., Astrup A. (2000). Reproducibility, power and validity of visual analogue scales in assessment of appetite sensations in single test meal studies. Int. J. Obes. Relat. Metab. Disord..

[4] Moher D., Liberati A., Tetzlaff J., Altman D.G., Group P. (2009). Preferred reporting items for systematic reviews and meta-analyses: The PRISMA statement. Ann. Intern. Med..

[5] Lluch A., Hanet-Geisen N., Salah S., Salas-Salvadó J., L’Heureux-Bouron D., Halford J.C. (2010). Short-term appetite-reducing effects of a low-fat dairy product enriched with protein and fibre. Food Qual. Preference.

[6] Schuring E., Quadt F., Kovacs E.M., Meullenet J.F., Wiseman S., Mela D.J. (2012). A quantitative method for estimating and comparing the duration of human satiety responses: Statistical modeling and application to liquid meal replacers. Appetite.

[7] Livingstone M.B.E., Robson P.J., Welch R.W., Burns A.A., Burrows M.S., McCormack C. (2000). Methodological issues in the assessment of satiety. Scand. J. Nutr..

[8] Higgins J.P., Altman D.G., Sterne J. (2008). Cochrane Handbook for Systematic Reviews of Interventions Version 5.1.0.

[9] Ibarra A., Astbury N.M., Olli K., Alhoniemi E., Tiihonen K. (2015). Effects of polydextrose on different levels of energy intake. A systematic review and meta-analysis. Appetite.

[10] Astbury N.M., Taylor M.A., French S.J., Macdonald I.A. (2014). Snacks containing whey protein and polydextrose induce a sustained reduction in daily energy intake over 2 weeks under free-living conditions. Am. J. Clin. Nutr..

[11] Olli K., Salli K., Alhoniemi E., Saarinen M., Ibarra A., Vasankari T., Rautonen N., Tiihonen K. (2015). Postprandial effects of polydextrose on satiety hormone responses and subjective feelings of appetite in obese participants. Nutr. J..

[12] Astbury N.M., Taylor M., Macdonald I.A. (2008). The effects of a polydextrose preload on appetite and energy intake. Proc. Nutr. Soc..

[13] Team R.D.C. (2013). R: A Language and Environment for Statistical Computing.

[14] Viechtbauer W. (2010). Conducting meta-analyses in R with the metafor package. J. Stat. Softw..

[15] Astbury N.M., Taylor M.A., Macdonald I.A. (2013). Polydextrose results in a dose-dependent reduction in *ad libitum* energy intake at a subsequent test meal. Br. J. Nutr..

[16] Ranawana V., Muller A., Henry C.J. (2013). Polydextrose: Its impact on short-term food intake and subjective feelings of satiety in males-a randomized controlled cross-over study. Eur. J. Nutr..

[17] Hull S., Re R., Tiihonen K., Viscione L., Wickham M. (2012). Consuming polydextrose in a mid-morning snack increases acute satiety measurements and reduces subsequent energy intake at lunch in healthy human subjects. Appetite.

[18] Schwab U., Louheranta A., Torronen A., Uusitupa M. (2006). Impact of sugar beet pectin and polydextrose on fasting and postprandial glycemia and fasting concentrations of serum total and lipoprotein lipids in middle-aged subjects with abnormal glucose metabolism. Eur. J. Clin. Nutr..

[19] King N.A., Craig S.A., Pepper T., Blundell J.E. (2005). Evaluation of the independent and combined effects of xylitol and polydextrose consumed as a snack on hunger and energy intake over 10 days. Br. J. Nutr..

[20] Hill A.J., Magson L.D., Blundell J.E. (1984). Hunger and palatability: Tracking ratings of subjective experience before, during and after the consumption of preferred and less preferred food. Appetite.

[21] Kissileff H.R., Klingsberg G., van Itallie T.B. (1980). Universal eating monitor for continuous recording of solid or liquid consumption in man. Am. J. Physiol..

[22] Yeomans M.R. (2000). Rating changes over the course of meals: What do they tell us about motivation to eat?. Neurosci. Biobehav. Rev..

[23] Delargy H., Lawton C., Smith F., King N., Blundell J. (1996). Electronic appetite rating system (EARS): Validation of continuous automated monitoring of motivation to eat. Int. J. Obes..

[24] Stubbs R.J., Hughes D.A., Johnstone A.M., Rowley E., Ferris S., Elia M., Stratton R., King N., Blundell J.E. (2001). Description and evaluation of a Newton-based electronic appetite rating system for temporal tracking of appetite in human subjects. Physiol. Behav..

[25] Cohen J. (1977). Statistical Power Analysis for the Behavioral Sciences.

[26] Slavin J., Green H. (2007). Dietary fibre and satiety. Nutr. Bull..

[27] (2009). Joint FAO/WHO Food Standards Programme Codex Alimentarius Commission. Proceedings of the 30th Session of the Codex Committee on Nutrition and Foods for Special Dietary Uses. Codex Alimentarius Commission Thirty Second Session—ALINORM 09/32/26.

[28] Craig S., Holden J., Troup J., Auerbach M., Frier H. (1998). Polydextrose as soluble fiber: Physiological and analytical aspects. Cereal Foods World (USA).

[29] Gregersen N.T., Møller B.K., Raben A., Kristensen S.T., Holm L., Flint A., Astrup A. (2011). Determinants of appetite ratings: The role of age, gender, BMI, physical activity, smoking habits, and diet/weight concern. Food Nutr. Res..

[30] Konings E., Schoffelen P.F., Stegen J., Blaak E.E. (2014). Effect of polydextrose and soluble maize fibre on energy metabolism, metabolic profile and appetite control in overweight men and women. Br. J. Nutr..

[31] Ravn A.-M., Gregersen N.T., Christensen R., Rasmussen L.G., Hels O., Belza A., Raben A., Larsen T.M., Toubro S., Astrup A. (2013). Thermic effect of a meal and appetite in adults: An individual participant data meta-analysis of meal-test trials. Food Nutr. Res..

[32] Green S., Delargy H., Joanes D., Blundell J. (1997). A satiety quotient: A formulation to assess the satiating effect of food. Appetite.

