# Supplementary material for: Effect of Polydextrose on Subjective Feelings of Appetite during the Satiation and Satiety Periods: A Systematic Review and Meta-Analysis
Source: Nutrients. 2016 Jan 14;8(1):45. doi: 10.3390/nu8010045 (PMC4728658; doi:10.3390/nu8010045)
Supplement: Supplementary file 1 [file nutrients-08-00045-s001.docx]

Supplementary Materials: Effect of Polydextrose on Subjective Feelings of Appetite during the Satiation and Satiety Periods: A Systematic Review and Meta-Analysis

Alvin Ibarra, Nerys M. Astbury, Kaisa Olli, Esa Alhoniemi and Kirsti Tiihonen

**Figure S1.** Example of the data adjustment to calculate incremental Areas under the Curve (iAUC) for Hunger, Prospective Food Consumption, and the Desire to Eat (I) during the Satiation period and (II) during the Satiety period for an ideal *verum* versus *placebo*.

**Figure S2.** Example of the data adjustment to calculate incremental Areas under the Curve (iAUC) for Satisfaction and Fullness (I) during the Satiation period and (II) during the Satiety period for an ideal *verum* versus *placebo*.


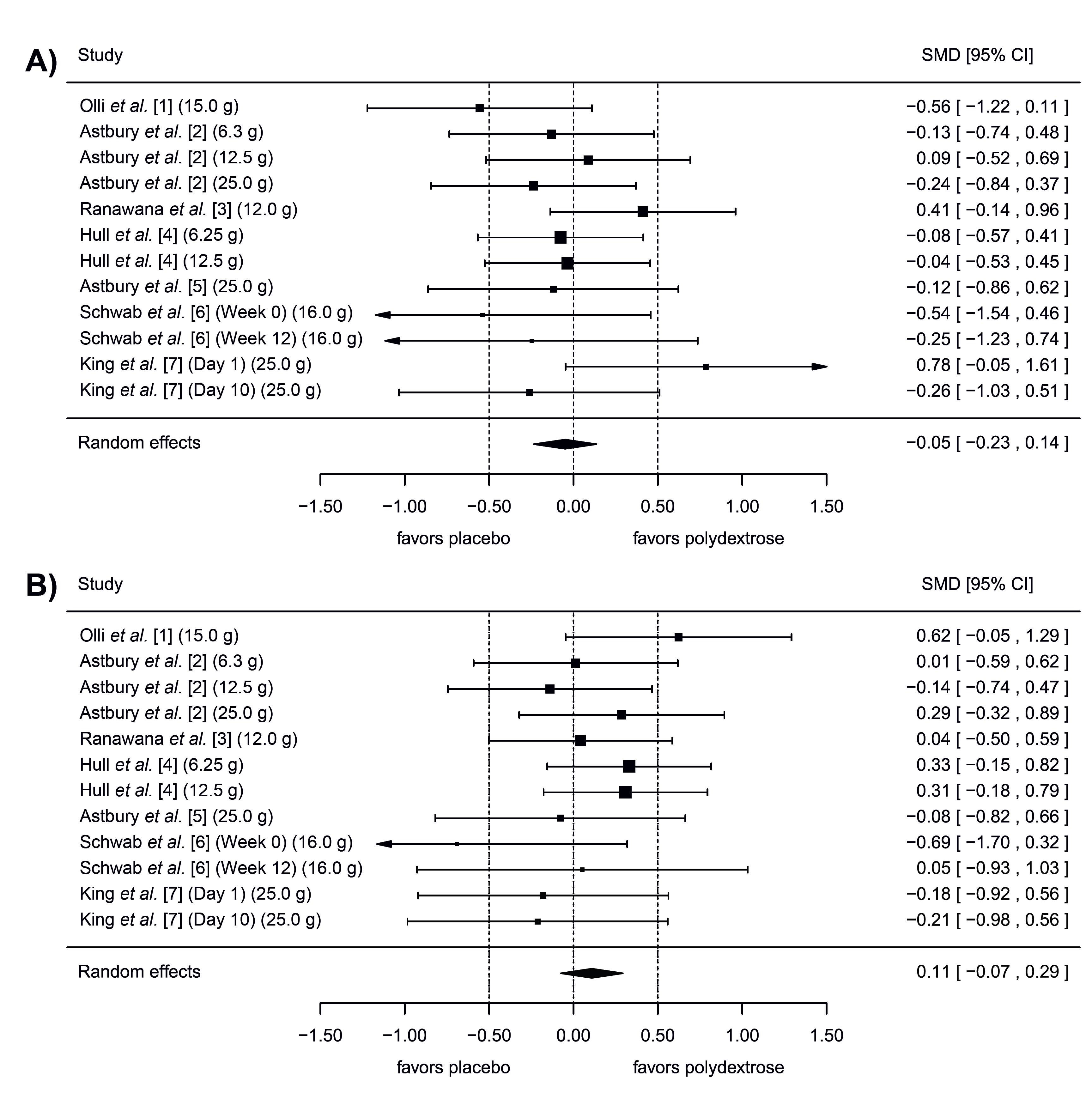


**Figure S3.** Meta-analysis comparing doses of polydextrose versus placebo in the studies included in the review on subjective feelings of Hunger, adjusted to show “less hungry with polydextrose” if the Standardized Mean Difference calculated using Hedges’ g measure (SMD, 95% CI) favors it, (**A**) during the Satiation period and (**B**) during the Satiety period. Doses of polydextrose per day used in each treatment are presented in brackets next to each reference.


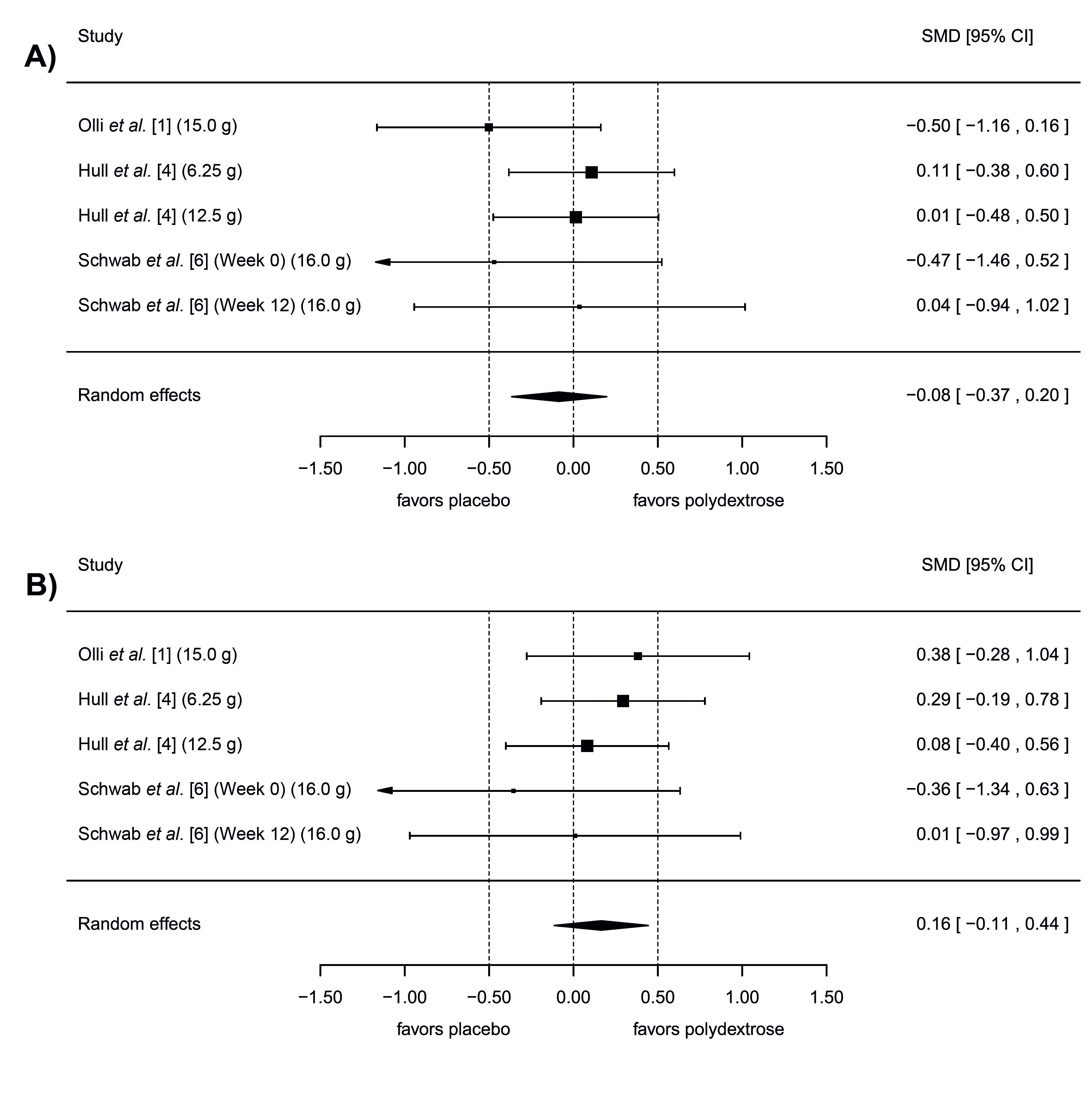


**Figure S4.** Meta-analysis comparing doses of polydextrose versus placebo in the studies included in the review on subjective feelings of Satisfaction, adjusted to show “more satisfied with polydextrose” if the Standardized Mean Difference calculated using Hedges’ g measure (SMD, 95% CI) favors it, (**A**) during the Satiation period and (**B**) during the Satiety period. Doses of polydextrose per day used in each treatment are presented in brackets next to each reference.


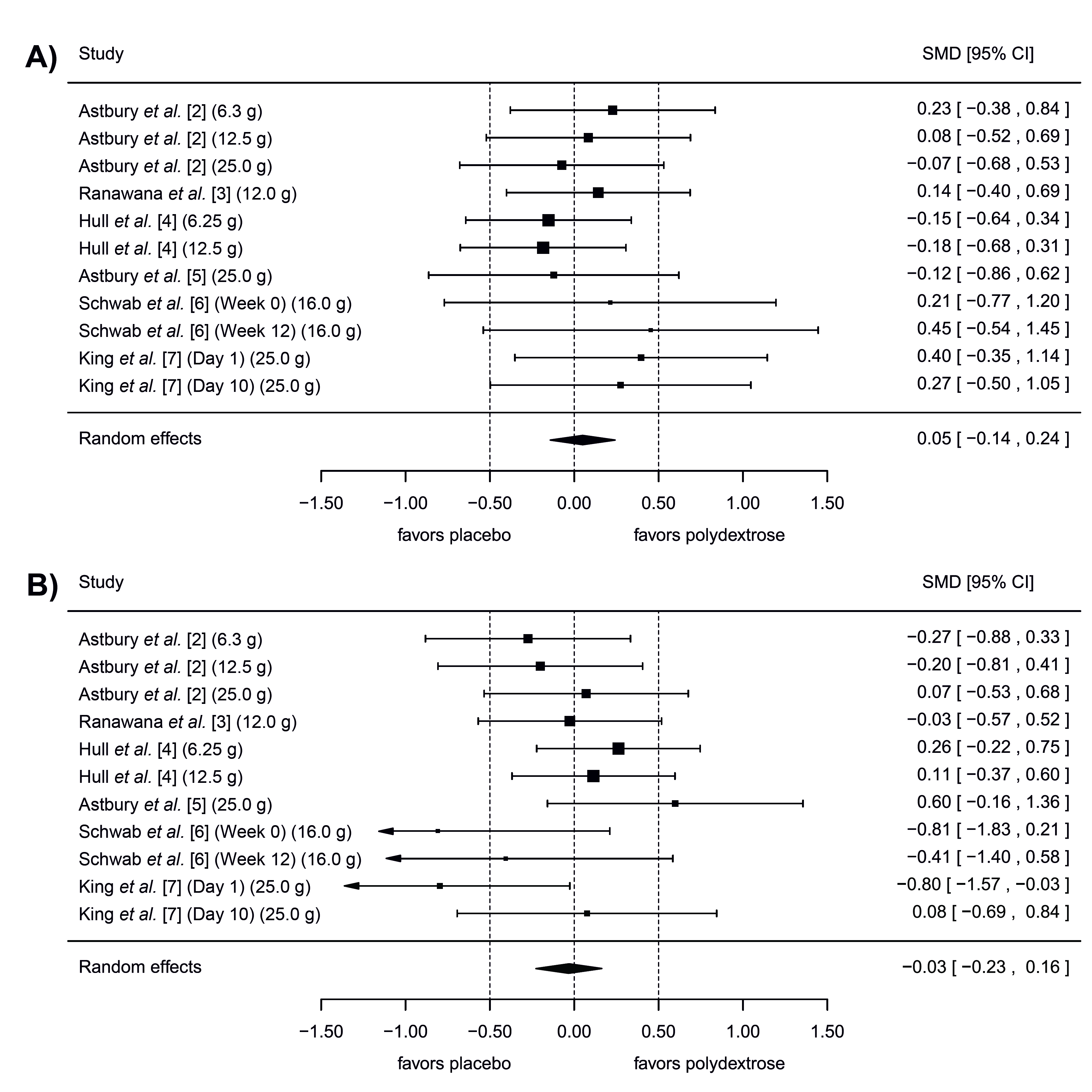


**Figure S5.** Meta-analysis comparing doses of polydextrose versus placebo in the studies included in the review on subjective feelings of Fullness, adjusted to show “more fullness with polydextrose” if the Standardized Mean Difference calculated using Hedges’ g measure (SMD, 95% CI) favors it,
(**A**) during the Satiation period and (**B**) during the Satiety period. Doses of polydextrose per day used in each treatment are presented in brackets next to each reference.


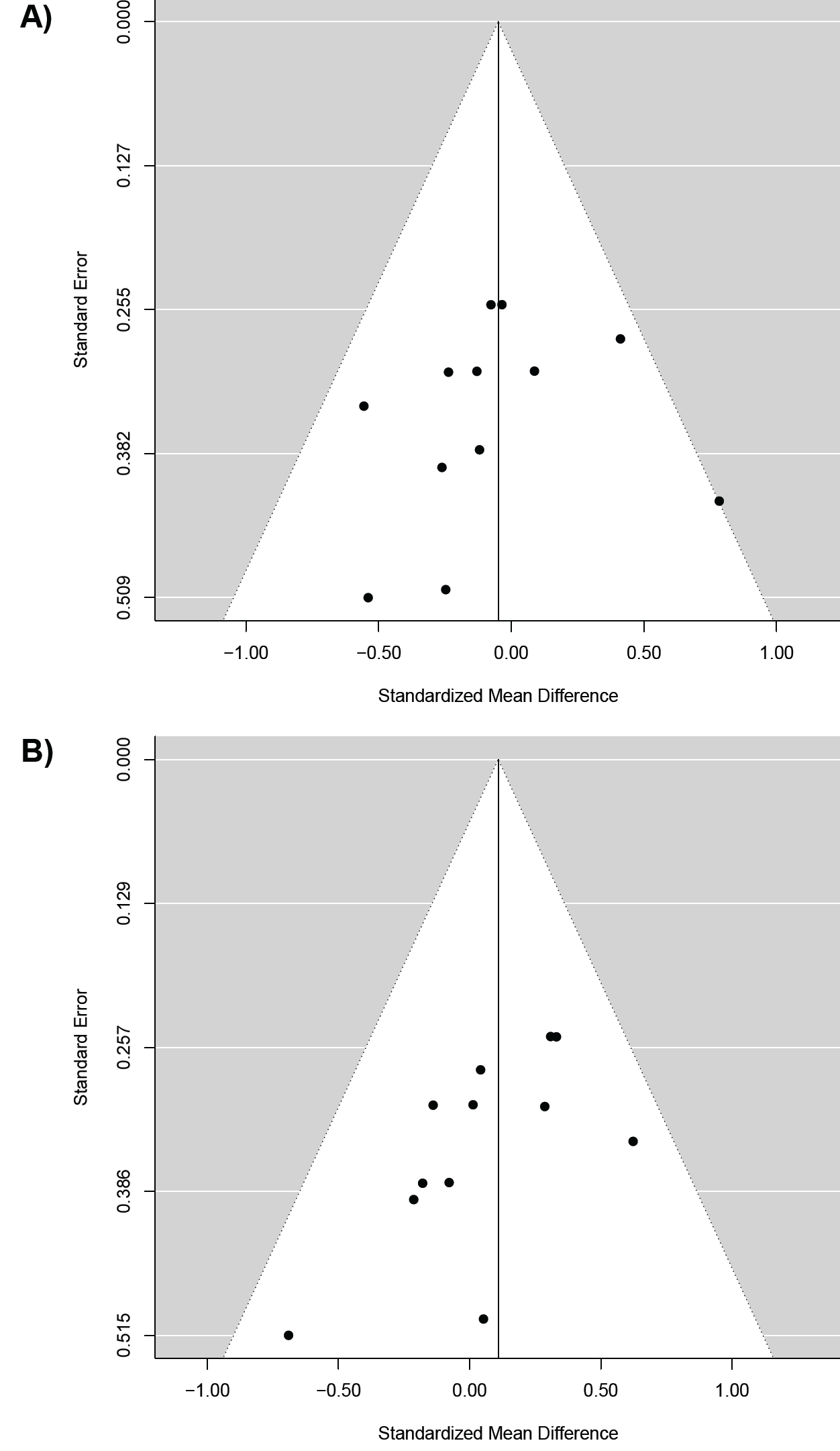


**Figure S6.** Funnel plot of the meta-analysis comparing doses of polydextrose versus placebo in the studies included in the review on subjective feelings of Hunger (**A**) during the Satiation period and (**B**) during the Satiety period.


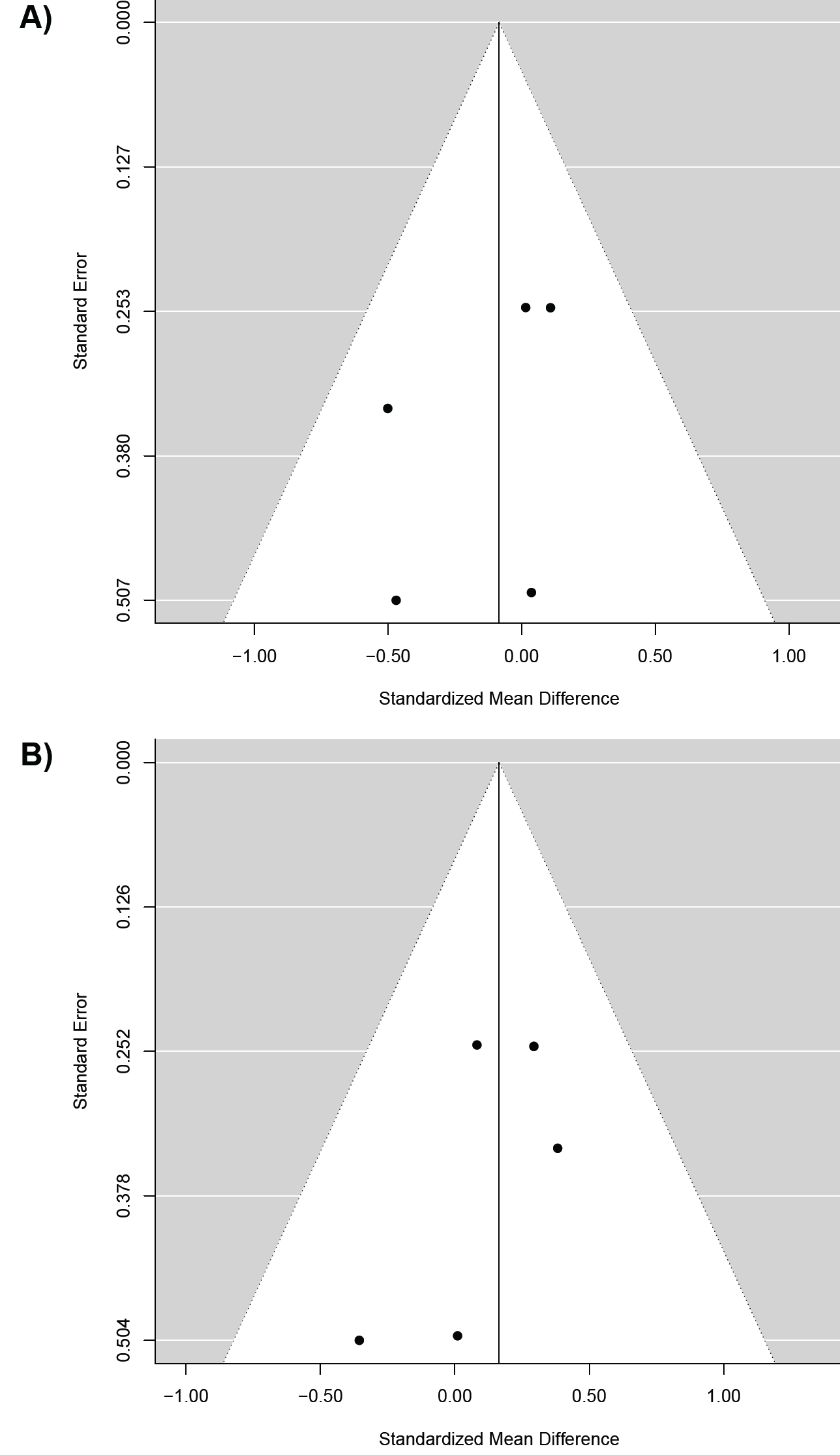


**Figure S7.** Funnel plot of the meta-analysis comparing doses of polydextrose versus placebo in the studies included in the review on subjective feelings of Satisfaction (**A**) during the Satiation period and (**B**) during the Satiety period.


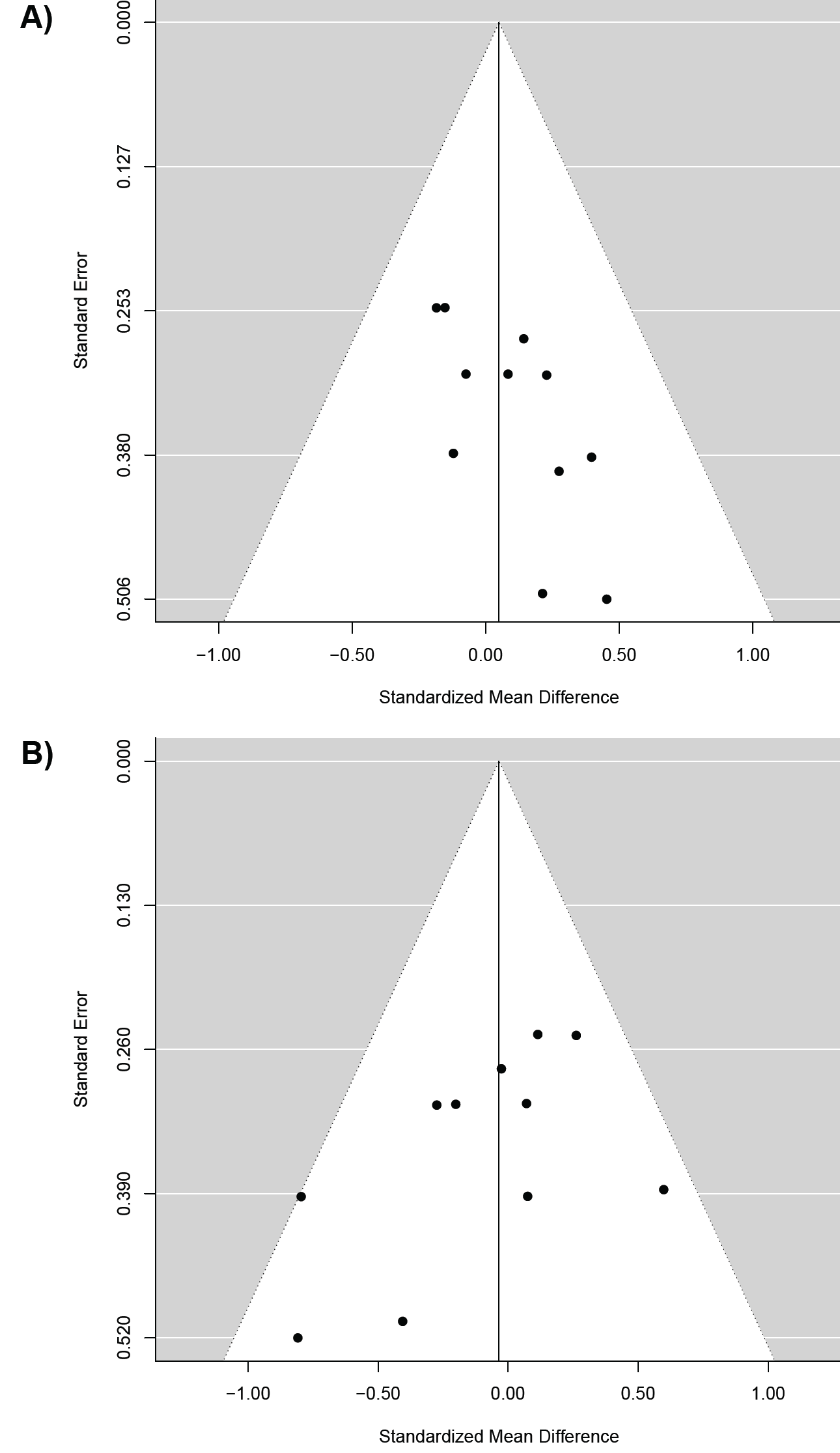


**Figure S8.** Funnel plot of the meta-analysis comparing doses of polydextrose versus placebo in the studies included in the review on subjective feelings of Fullness (**A**) during the Satiation period and (**B**) during the Satiety period.


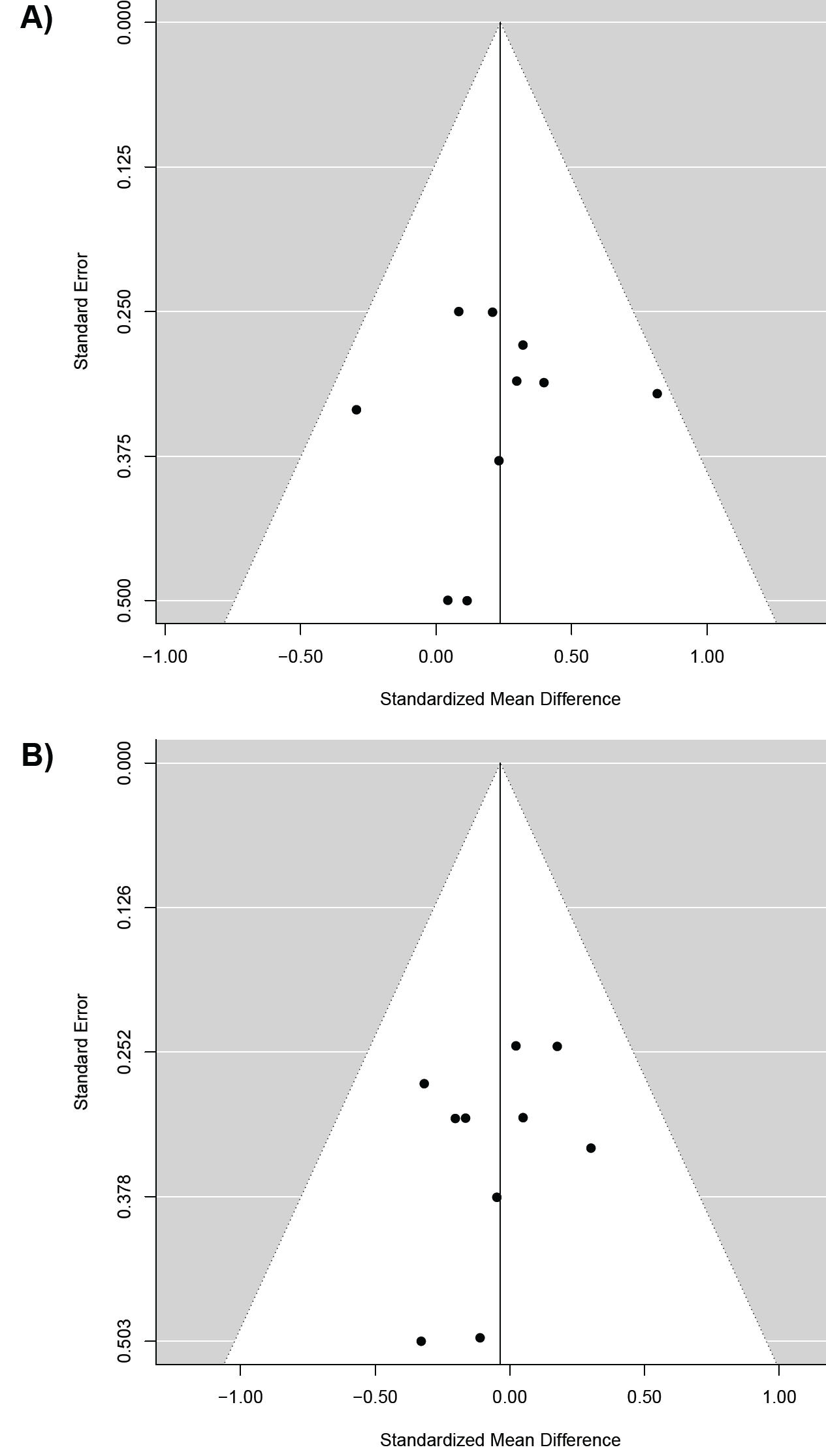


**Figure S9.** Funnel plot of the meta-analysis comparing doses of polydextrose versus placebo in the studies included in the review on subjective feelings of Desire to Eat (**A**) during the Satiation period and (**B**) during the Satiety period.

**Table S1.** Generic search strategy to identify studies reporting the effects of polydextrose on subjective feelings of appetite and energy intake levels.

| **Criterion Number** | **Key Words** |
| --- | --- |
| 1 | Polydextrose |
| 2 | Appetite |
| 3 | Hunger |
| 4 | Satiety |
| 5 | Fullness |
| 6 | Prospective food consumption |
| 7 | Desire to eat |
| 8 | 1 AND 2, OR 3, 4, 5, 6, 7 |
| 9 | Energy intake |
| 10 | Caloric intake |
| 11 | Dietary intake |
| 12 | Food intake |
| 13 | 1 AND 9, OR 10, 11, 12 |

**Table S2.** Number of hits per database searched.

| **Database** | **Number of Hits** |
| --- | --- |
| Medline | 139485 |
| Proquest | 693754 |
| ClinicalTrials.Gov | 6785 |
| Science Direct | 419961 |
| Wiley Online Library | 249925 |
| Other Sources | 13 |
| Total Hits | **1509923** |

**Table S3.** Hunger: Incremental Areas under the Curve during Satiation (iAUC_Satiation_) and Satiety (iAUC_Satiety_) periods, and their relative changes in percentage, for the placebo and polydextrose treatments of the studies included in the meta-analysis.

| **Studies** | **Polydextrose (g)** | ***n*** | **iAUC_Satiation_ (min·mm) ^a^** | | ***n*** | **iAUC_Satiety_ (min·mm)** | |
| --- | --- | --- | --- | --- | --- | --- | --- |
|  |  |  | **Placebo (Mean ± SD)** | **Polydextrose (Mean ± SD)** |  | **Placebo (Mean ± SD)** | **Polydextrose (Mean ± SD)** |
| **Whole group** |  |  |  |  |  |  |  |
| Olli *et al.* [1] | 15.0 | 18 | −1176 ± 571 | −794 ± 757 | 18 | 5487 ± 2969 | 3272 ± 3917 |
| Astbury *et al.* [2] | 6.3 | 21 | −426 ± 192 | −397 ± 252 | 21 | 3759 ± 1780 | 3734 ± 1983 |
| Astbury *et al.* [2] | 12.5 | 21 | −426 ± 192 | −444 ± 210 | 21 | 3759 ± 1780 | 4021 ± 1913 |
| Astbury *et al.* [2] | 25.0 | 21 | −426 ± 192 | −375 ± 228 | 21 | 3759 ± 1780 | 3205 ± 2013 |
| Ranawana *et al.* [3] | 12.0 | 26 | −114 ± 228 | −192 ± 131 | 26 | 462 ± 562 | 439 ± 555 |
| Hull *et al.* [4] | 6.25 | 32 | −156 ± 117 | −145 ± 145 | 33 | 1319 ± 1174 | 946 ± 1047 |
| Hull *et al.* [4] | 12.5 | 32 | −156 ± 117 | −151 ± 147 | 33 | 1319 ± 1174 | 973 ± 1031 |
| Astbury *et al.* [5] | 25.0 | 14 | −201 ± 173 | −182 ± 119 | 14 | 1748 ± 2351 | 1902 ± 1334 |
| Schwab *et al.* [6] (week 0) | 16.0 | 8 | −120 ± 178 | −37 ± 106 | 8 | 1165 ± 4123 | 3507 ± 1891 |
| Schwab *et al.* [6] (week 12) | 16.0 | 8 | −95 ± 162 | −61 ± 79 | 8 | 1602 ± 5829 | 1333 ± 3552 |
| King *et al.* [7] (day 1) | 25.0 | 12 | −69 ± 77 | −143 ± 103 | 14 | 924 ± 1044 | 984 ± 928 |
| King *et al.* [7] (day 10) | 25.0 | 13 | −91 ± 144 | −58 ± 94 | 14 | 798 ± 732 | 924 ± 674 |
| **Males** |  |  |  |  |  |  |  |
| Olli *et al.* [1] | 15.0 | 5 | −1220 ± 688 | −580 ± 509 | 5 | 4382 ± 3105 | 792 ± 3141 |
| Astbury *et al.* [2] | 6.3 | 12 | −461 ± 157 | −439 ± 246 | 12 | 3936 ± 1723 | 4261 ± 1790 |
| Astbury *et al.* [2] | 12.5 | 12 | −461 ± 157 | −516 ± 161 | 12 | 3936 ± 1723 | 4344 ± 1889 |
| Astbury *et al.* [2] | 25.0 | 12 | −461 ± 157 | −395 ± 250 | 12 | 3936 ± 1723 | 3404 ± 1837 |
| Ranawana *et al.* [3] | 12.0 | 26 | −114 ± 228 | −192 ± 131 | 26 | 462 ± 562 | 439 ± 555 |
| Hull *et al.* [4] | 6.25 | 8 | −116 ± 83 | −89 ± 111 | 9 | 986 ± 723 | 944 ± 661 |
| Hull *et al.* [4] | 12.5 | 8 | −116 ± 83 | −133 ± 123 | 9 | 986 ± 723 | 638 ± 598 |
| Astbury *et al.* [5] | 25.0 | 14 | −201 ± 173 | −182 ± 119 | 14 | 1748 ± 2351 | 1902 ± 1334 |
| Schwab *et al.* [6] (week 0) | 16.0 | 2 | −49 ± 207 | 71 ± 80 | 2 | 4316 ± 1013 | 1770 ± 308 |
| Schwab *et al.* [6] (week 12) | 16.0 | 2 | −98 ± 85 | −68 ± 11 | 2 | 4241 ± 5956 | 3855 ± 4423 |
| King *et al.* [7] (day 1) | 25.0 | 7 | −47 ± 77 | −177 ± 136 | 7 | 691 ± 762 | 885 ± 1046 |
| King *et al.* [7] (day 10) | 25.0 | 6 | −64 ± 171 | −66 ± 116 | 7 | 732 ± 697 | 722 ± 380 |

**Table S3.** *Cont.*

| **Studies** | **Polydextrose (g)** | ***n*** | **iAUC_Satiation_ (min·mm) ^a^** | | ***n*** | **iAUC_Satiety_ (min·mm)** | |
| --- | --- | --- | --- | --- | --- | --- | --- |
|  |  |  | **Placebo (Mean ± SD)** | **Polydextrose (Mean ± SD)** |  | **Placebo (Mean ± SD)** | **Polydextrose (Mean ± SD)** |
| **Females** |  |  |  |  |  |  |  |
| Olli *et al.* [1] | 15.0 | 13 | −1158 ± 550 | −877 ± 837 | 13 | 5912 ± 2927 | 4226 ± 3860 |
| Astbury *et al.* [2] | 6.3 | 9 | −380 ± 232 | −340 ± 263 | 9 | 3524 ± 1932 | 3032 ± 2110 |
| Astbury *et al.* [2] | 12.5 | 9 | −380 ± 232 | −348 ± 238 | 9 | 3524 ± 1932 | 3591 ± 1968 |
| Astbury *et al.* [2] | 25.0 | 9 | −380 ± 232 | −350 ± 208 | 9 | 3524 ± 1932 | 2939 ± 2313 |
| Hull *et al.* [4] | 6.25 | 24 | −169 ± 125 | −169 ± 154 | 24 | 1443 ± 1295 | 947 ± 1184 |
| Hull *et al.* [4] | 12.5 | 24 | −169 ± 125 | −161 ± 160 | 24 | 1443 ± 1295 | 1156 ± 1177 |
| Schwab *et al.* [6] (week 0) | 16.0 | 6 | −144 ± 182 | −73 ± 90 | 6 | 115 ± 4278 | 4086 ± 1837 |
| Schwab *et al.* [6] (week 12) | 16.0 | 6 | −94 ± 188 | −59 ± 93 | 6 | 723 ± 6063 | 492 ± 3219 |
| King *et al.* [7] (day 1) | 25.0 | 5 | −99 ± 73 | −118 ± 73 | 7 | 1157 ± 1286 | 1082 ± 865 |
| King *et al.* [7] (day 10) | 25.0 | 7 | −114 ± 126 | −45 ± 61 | 7 | 865 ± 816 | 1206 ± 929 |

g = grams. iAUC = incremental area under the curve. min = minute. mm = millimeter. *n* = number of participants. SD = standard deviation. ^a^ values in the Satiation period have been multiplied by −1 to indicate that negative values “reduce hunger”.

**Table S4.** Satisfaction: Incremental Areas under the Curve during Satiation (iAUC_Satiation_) and Satiety (iAUC_Satiety_) periods, and their relative changes in percentage, for the placebo and polydextrose treatments of the studies included in the meta-analysis.

| **Studies** | **Polydextrose (g)** | ***n*** | **iAUC_Satiation_ (min·mm)** | | ***n*** | **iAUC_Satiety_ (min·mm) ^a^** | |
| --- | --- | --- | --- | --- | --- | --- | --- |
|  |  |  | **Placebo (Mean ± SD)** | **Polydextrose (Mean ± SD)** |  | **Placebo (Mean ± SD)** | **Polydextrose (Mean ± SD)** |
| **Whole group** |  |  |  |  |  |  |  |
| Olli *et al.* [1] | 15.0 | 18 | −1409 ± 403 | −1144 ± 608 | 18 | 6944 ± 4644 | 5385 ± 3195 |
| Hull *et al.* [4] | 6.25 | 32 | −108 ± 104 | −122 ± 152 | 33 | 791 ± 1005 | 504 ± 925 |
| Hull *et al.* [4] | 12.5 | 32 | −108 ± 104 | −110 ± 133 | 33 | 791 ± 1005 | 703 ± 1113 |
| Schwab *et al.* [6] (week 0) | 16.0 | 8 | −209 ± 158 | −85 ± 315 | 8 | 817 ± 4308 | 2627 ± 5268 |
| Schwab *et al.* [6] (week 12) | 16.0 | 8 | −137 ± 76 | −144 ± 247 | 8 | 1648 ± 3948 | 1614 ± 1967 |
| **Males** |  |  |  |  |  |  |  |
| Olli *et al.* [1] | 15.0 | 5 | −1376 ± 342 | −1048 ± 548 | 5 | 5138 ± 3833 | 4660 ± 1983 |
| Hull *et al.* [4] | 6.25 | 8 | −92 ± 84 | −108 ± 130 | 9 | 953 ± 691 | 802 ± 679 |
| Hull *et al.* [4] | 12.5 | 8 | −92 ± 84 | −110 ± 131 | 9 | 953 ± 691 | 438 ± 489 |
| Schwab *et al.* [6] (week 0) | 16.0 | 2 | −296 ± 69 | −311 ± 217 | 2 | 4436 ± 886 | 3998 ± 859 |
| Schwab *et al.* [6] (week 12) | 16.0 | 2 | −184 ± 16 | −146 ± 16 | 2 | 1549 ± 1755 | 2561 ± 2986 |
| **Females** |  |  |  |  |  |  |  |
| Olli *et al.* [1] | 15.0 | 13 | −1422 ± 437 | −1182 ± 647 | 13 | 7639 ± 4876 | 5664 ± 3585 |
| Hull *et al.* [4] | 6.25 | 24 | −113 ± 110 | −128 ± 162 | 24 | 730 ± 1107 | 380 ± 996 |
| Hull *et al.* [4] | 12.5 | 24 | −113 ± 110 | −110 ± 137 | 24 | 730 ± 1107 | 848 ± 1326 |
| Schwab *et al.* [6] (week 0) | 16.0 | 6 | −180 ± 173 | −10 ± 319 | 6 | −390 ± 4340 | 2170 ± 6140 |
| Schwab *et al.* [6] (week 12) | 16.0 | 6 | −121 ± 83 | −143 ± 292 | 6 | 1681 ± 4605 | 1299 ± 1776 |

g = grams. iAUC = incremental area under the curve. min = minute. mm = millimeter. *n* = number of participants. SD = standard deviation. ^a^ values in the Satiety period have been multiplied by −1 to indicate that positive values.

**Table S5.** Fullness: Incremental Areas under the Curve during Satiation (iAUC_Satiation_) and Satiety (iAUC_Satiety_) periods, and their relative changes in percentage, for the placebo and polydextrose treatments of the studies included in the meta-analysis.

| **Studies** | **Polydextrose (g)** | ***n*** | **iAUC_Satiation_ (min·mm)** | | ***n*** | **iAUC_Satiety_ (min·mm) ^a^** | |
| --- | --- | --- | --- | --- | --- | --- | --- |
|  |  |  | **Placebo (Mean ± SD)** | **Polydextrose (Mean ± SD)** |  | **Placebo (Mean±SD)** | **Polydextrose (Mean ± SD)** |
| **Whole group** |  |  |  |  |  |  |  |
| Astbury *et al.* [2] | 6.3 | 21 | −255 ± 183 | −300 ± 200 | 21 | 1625 ± 1788 | 2108 ± 1666 |
| Astbury *et al.* [2] | 12.5 | 21 | −255 ± 183 | −271 ± 195 | 21 | 1625 ± 1788 | 2029 ± 2136 |
| Astbury *et al.* [2] | 25.0 | 21 | −255 ± 183 | −240 ± 211 | 21 | 1625 ± 1788 | 1500 ± 1686 |
| Ranawana *et al.* [3] | 12.0 | 26 | −164 ± 216 | −190 ± 128 | 26 | 414 ± 469 | 427 ± 543 |
| Hull *et al.* [4] | 6.25 | 32 | −131 ± 123 | −111 ± 136 | 33 | 926 ± 1300 | 624 ± 950 |
| Hull *et al.* [4] | 12.5 | 32 | −131 ± 123 | −106 ± 141 | 33 | 926 ± 1300 | 791 ± 1005 |
| Astbury *et al.* [5] | 25.0 | 14 | −275 ± 177 | −255 ± 150 | 14 | 2513 ± 1117 | 1791 ± 1225 |
| Schwab *et al.* [6] (week 0) | 16.0 | 8 | −199 ± 194 | −248 ± 236 | 8 | 1118 ± 4574 | 4631 ± 3583 |
| Schwab *et al.* [6] (week 12) | 16.0 | 8 | −123 ± 102 | −215 ± 251 | 8 | 987 ± 4196 | 2425 ± 2205 |
| King *et al.* [7] (day 1) | 25.0 | 14 | −91 ± 158 | −149 ± 123 | 14 | 503 ± 930 | 1104 ± 922 |
| King *et al.* [7] (day 10) | 25.0 | 13 | −23 ± 169 | −66 ± 137 | 14 | 662 ± 716 | 639 ± 815 |
| **Males** |  |  |  |  |  |  |  |
| Astbury *et al.* [2] | 6.3 | 12 | −267 ± 188 | −349 ± 209 | 12 | 1586 ± 1906 | 2829 ± 1708 |
| Astbury *et al.* [2] | 12.5 | 12 | −267 ± 188 | −256 ± 228 | 12 | 1586 ± 1906 | 1773 ± 2449 |
| Astbury *et al.* [2] | 25.0 | 12 | −267 ± 188 | −237 ± 132 | 12 | 1586 ± 1906 | 1415 ± 1668 |
| Ranawana *et al.* [3] | 12.0 | 26 | −164 ± 216 | −190 ± 128 | 26 | 414 ± 469 | 427 ± 543 |
| Hull *et al.* [4] | 6.25 | 8 | −91 ± 71 | −57 ± 101 | 9 | 848 ± 695 | 734 ± 827 |
| Hull *et al.* [4] | 12.5 | 8 | −91 ± 71 | −100 ± 152 | 9 | 848 ± 695 | 527 ± 627 |
| Astbury *et al.* [5] | 25.0 | 14 | −275 ± 177 | −255 ± 150 | 14 | 2513 ± 1117 | 1791 ± 1225 |
| Schwab *et al.* [6] (week 0) | 16.0 | 2 | −289 ± 69 | −206 ± 37 | 2 | 4639 ± 1024 | 1755 ± 1888 |
| Schwab *et al.* [6] (week 12) | 16.0 | 2 | −169 ± 48 | −146 ± 37 | 2 | 859 ± 811 | 1894 ± 1692 |
| King *et al.* [7] (day 1) | 25.0 | 7 | −79 ± 60 | −179 ± 167 | 7 | 618 ± 1182 | 995 ± 1162 |
| King *et al.* [7] (day 10) | 25.0 | 6 | −38 ± 152 | −130 ± 86 | 7 | 516 ± 719 | 796 ± 748 |

**Table S5.** *Cont.*

| **Studies** | **Polydextrose (g)** | ***n*** | **iAUC_Satiation_ (min·mm)** | | ***n*** | **iAUC_Satiety_ (min·mm) ^a^** | |
| --- | --- | --- | --- | --- | --- | --- | --- |
|  |  |  | **Placebo (Mean ± SD)** | **Polydextrose (Mean ± SD)** |  | **Placebo (Mean±SD)** | **Polydextrose (Mean ± SD)** |
| **Females** |  |  |  |  |  |  |  |
| Astbury *et al.* [2] | 6.3 | 9 | −239 ± 186 | −234 ± 178 | 9 | 1678 ± 1730 | 1148 ± 1055 |
| Astbury *et al.* [2] | 12.5 | 9 | −239 ± 186 | −293 ± 152 | 9 | 1678 ± 1730 | 2370 ± 1713 |
| Astbury *et al.* [2] | 25.0 | 9 | −239 ± 186 | −244 ± 296 | 9 | 1678 ± 1730 | 1613 ± 1806 |
| Hull *et al.* [4] | 6.25 | 24 | −144 ± 135 | −133 ± 144 | 24 | 955 ± 1477 | 578 ± 1010 |
| Hull *et al.* [4] | 12.5 | 24 | −144 ± 135 | −110 ± 138 | 24 | 955 ± 1477 | 936 ± 1148 |
| Schwab *et al.* [6] (week 0) | 16.0 | 6 | −169 ± 218 | −261 ± 278 | 6 | −56 ± 4740 | 5590 ± 3584 |
| Schwab *et al.* [6] (week 12) | 16.0 | 6 | −108 ± 114 | −238 ± 292 | 6 | 1030 ± 4950 | 2602 ± 2467 |
| King *et al.* [7] (day 1) | 25.0 | 7 | −103 ± 224 | −128 ± 87 | 7 | 387 ± 667 | 1212 ± 682 |
| King *et al.* [7] (day 10) | 25.0 | 7 | −10 ± 193 | 11 ± 157 | 7 | 807 ± 738 | 455 ± 921 |

g = grams. iAUC = incremental area under the curve. min = minute. mm = millimeter. *n* = number of participants. SD = standard deviation. ^a^ values in the Satiety period have been multiplied by −1 to indicate that positive values “increase fullness”.

**Table S6.** Prospective Food Consumption: Incremental Areas under the Curve during Satiation (iAUC_Satiation_) and Satiety (iAUC_Satiety_) periods, and their relative changes in percentage, for the placebo and polydextrose treatments of the studies included in the meta-analysis.

| **Studies** | **Polydextrose (g)** | ***n*** | **iAUC_Satiation_ (min·mm) ^a^** | | ***n*** | **iAUC_Satiety_ (min·mm)** | |
| --- | --- | --- | --- | --- | --- | --- | --- |
|  |  |  | **Placebo (Mean ± SD)** | **Polydextrose (Mean ± SD)** |  | **Placebo (mean ± SD)** | **Polydextrose (Mean ± SD)** |
| **Whole group** |  |  |  |  |  |  |  |
| Ranawana *et al.* [3] | 12.0 | 26 | −96 ± 183 | −135 ± 99 | 26 | −394 ± 439 | −348 ± 501 |
| Hull *et al.* [4] | 6.25 | 32 | 128 ± 125 | 133 ± 130 | 33 | 1001 ± 988 | 911 ± 954 |
| Hull *et al.* [4] | 12.5 | 32 | 128 ± 125 | 121 ± 142 | 33 | 1001 ± 988 | 751 ± 1086 |
| **Males** |  |  |  |  |  |  |  |
| Ranawana *et al.* [3] | 12.0 | 26 | −96 ± 183 | −135 ± 99 | 26 | −394 ± 439 | −348 ± 501 |
| Hull *et al.* [4] | 6.25 | 8 | 92 ± 104 | 95 ± 110 | 9 | 954 ± 660 | 775 ± 796 |
| Hull *et al.* [4] | 12.5 | 8 | 92 ± 104 | 89 ± 129 | 9 | 954 ± 660 | 268 ± 668 |
| **Females** |  |  |  |  |  |  |  |
| Hull *et al.* [4] | 6.25 | 24 | 140 ± 131 | 148 ± 137 | 24 | 1019 ± 1098 | 968 ± 1023 |
| Hull *et al.* [4] | 12.5 | 24 | 140 ± 131 | 139 ± 149 | 24 | 1019 ± 1098 | 1015 ± 1189 |

g = grams. iAUC = incremental area under the curve. min = minute. mm = millimeter. *n* = number of participants. SD = standard deviation. ^a^ values in the Satiation period have been multiplied by −1 to indicate that negative values “lower the amount expected to eat”.

**Table S7.** Desire to Eat: Incremental Areas under the Curve during Satiation (iAUC_Satiation_) and Satiety (iAUC_Satiety_) periods, and their relative changes in percentage, for the placebo and polydextrose treatments of the studies included in the meta-analysis.

| **Studies** | **Polydextrose (g)** | ***n*** | **iAUC_Satiation_ (min·mm) ^a^** | | ***n*** | **iAUC_Satiety_ (min·mm)** | |
| --- | --- | --- | --- | --- | --- | --- | --- |
|  |  |  | **Placebo (Mean ± SD)** | **Polydextrose (Mean ± SD)** |  | **Placebo (Mean ± SD)** | **Polydextrose (Mean ± SD)** |
| **Whole group** |  |  |  |  |  |  |  |
| Olli *et al.* [1] | 15.0 | 18 | −1218 ± 570 | −1034 ± 648 | 18 | 6167 ± 4461 | 4939 ± 3453 |
| Astbury *et al.* [2] | 6.3 | 21 | −32 ± 330 | −149 ± 243 | 21 | 754 ± 2189 | 1127 ± 2254 |
| Astbury *et al.* [2] | 12.5 | 21 | −32 ± 330 | −281 ± 267 | 21 | 754 ± 2189 | 1192 ± 2050 |
| Astbury *et al.* [2] | 25.0 | 21 | −32 ± 330 | −122 ± 264 | 21 | 754 ± 2189 | 654 ± 1844 |
| Ranawana *et al.* [3] | 12.0 | 26 | −105 ± 213 | −161 ± 123 | 26 | 292 ± 492 | 468 ± 597 |
| Hull *et al.* [4] | 6.25 | 32 | −146 ± 138 | −174 ± 130 | 33 | 1123 ± 1277 | 923 ± 948 |
| Hull *et al.* [4] | 12.5 | 32 | −146 ± 138 | −159 ± 173 | 33 | 1123 ± 1277 | 1097 ± 971 |
| Astbury *et al.* [5] | 25.0 | 14 | 66 ± 273 | 11 ± 179 | 14 | 1381 ± 3190 | 1527 ± 2649 |
| Schwab *et al.* [6] (week 0) | 16.0 | 8 | −220 ± 211 | −230 ± 199 | 8 | 1014 ± 5491 | 2458 ± 2049 |
| Schwab *et al.* [6] (week 12) | 16.0 | 8 | −105 ± 197 | −124 ± 95 | 8 | 1192 ± 5315 | 1696 ± 2980 |
| **Males** |  |  |  |  |  |  |  |
| Olli *et al.* [1] | 15.0 | 5 | −1220 ± 680 | −900 ± 575 | 5 | 5228 ± 2741 | 3378 ± 2598 |
| Astbury *et al.* [2] | 6.3 | 12 | 11 ± 288 | −172 ± 226 | 12 | 739 ± 1941 | 1071 ± 2385 |
| Astbury *et al.* [2] | 12.5 | 12 | 11 ± 288 | −207 ± 251 | 12 | 739 ± 1941 | 468 ± 1852 |
| Astbury *et al.* [2] | 25.0 | 12 | 11 ± 288 | −148 ± 253 | 12 | 739 ± 1941 | 357 ± 2009 |
| Ranawana *et al.* [3] | 12.0 | 26 | −105 ± 213 | −161 ± 123 | 26 | 292 ± 492 | 468 ± 597 |
| Hull *et al.* [4] | 6.25 | 8 | −109 ± 152 | −110 ± 95 | 9 | 738 ± 833 | 833 ± 679 |
| Hull *et al.* [4] | 12.5 | 8 | −109 ± 152 | −127 ± 127 | 9 | 738 ± 833 | 660 ± 559 |
| Astbury *et al.* [5] | 25.0 | 14 | 66 ± 273 | 11 ± 179 | 14 | 1381 ± 3190 | 1527 ± 2649 |
| Schwab *et al.* [6] (week 0) | 16.0 | 2 | −158 ± 361 | −83 ± 159 | 2 | 5089 ± 1278 | 2231 ± 2265 |
| Schwab *et al.* [6] (week 12) | 16.0 | 2 | −15 ± 170 | −195 ± 148 | 2 | 2460 ± 4073 | 2738 ± 2493 |
| **Females** |  |  |  |  |  |  |  |
| Olli *et al.* [1] | 15.0 | 13 | −1217 ± 553 | −1086 ± 688 | 13 | 6528 ± 5018 | 5539 ± 3638 |
| Astbury *et al.* [2] | 6.3 | 9 | −88 ± 389 | −119 ± 275 | 9 | 775 ± 2608 | 1202 ± 2206 |
| Astbury *et al.* [2] | 12.5 | 9 | −88 ± 389 | −380 ± 267 | 9 | 775 ± 2608 | 2159 ± 1989 |
| Astbury *et al.* [2] | 25.0 | 9 | −88 ± 389 | −88 ± 290 | 9 | 775 ± 2608 | 1049 ± 1626 |
| Hull *et al.* [4] | 6.25 | 24 | −158 ± 134 | −201 ± 135 | 24 | 1268 ± 1395 | 961 ± 1050 |
| Hull *et al.* [4] | 12.5 | 24 | −158 ± 134 | −177 ± 194 | 24 | 1268 ± 1395 | 1336 ± 1072 |
| Schwab *et al.* [6] (week 0) | 16.0 | 6 | −241 ± 186 | −279 ± 196 | 6 | −344 ± 5747 | 2534 ± 2197 |
| Schwab *et al.* [6] (week 12) | 16.0 | 6 | −135 ± 211 | −100 ± 75 | 6 | 769 ± 5948 | 1349 ± 3258 |

g = grams. iAUC = incremental area under the curve. min = minute. mm = millimeter. *n* = number of participants. SD = standard deviation. ^a^ values in the Satiation period have been multiplied by −1 to indicate that negative values “reduce the desire to eat”.

**Table S8.** Model diagnostics for the meta-analysis models. The number of effect sizes (*K*), study heterogeneity assessed using *Q*-value (*Q*), study heterogeneity assessed using Higgins *I^2^* statistic (*I^2^*), intercept of the model used in the Egger’s test, and the *p*-value of Egger’s test.

| **Groups** | ***K*** | ***Q*** | ***I*^2^** | **Egger’s Test Intercept** | **Egger’s Test *p*-Value** |
| --- | --- | --- | --- | --- | --- |
| **Whole group** |  |  |  |  |  |
| Satiation |  |  |  |  |  |
| Hunger | 12 | 10.85 | 0 | 0.20 | 0.58 |
| Satisfaction | 5 | 2.90 | 0 | 0.35 | 0.35 |
| Fullness | 11 | 4.24 | 0 | −0.56 | 0.021 (*) |
| Desire to Eat | 10 | 6.75 | 0 | 0.34 | 0.81 |
| Satiety |  |  |  |  |  |
| Hunger | 12 | 8.77 | 0 | 0.86 | 0.040 (*) |
| Satisfaction | 5 | 1.97 | 0 | 0.53 | 0.33 |
| Fullness | 11 | 12.08 | 0.007 | 0.71 | 0.10 |
| Desire to Eat | 10 | 3.73 | 0 | 0.21 | 0.42 |
| **Males** |  |  |  |  |  |
| Satiation |  |  |  |  |  |
| Hunger | 12 | 9.59 | 0 | 0.50 | 0.26 |
| Satisfaction | 5 | 2.46 | 0 | 0.84 | 0.22 |
| Fullness | 11 | 5.63 | 0 | 0.21 | 0.69 |
| Desire to Eat | 10 | 4.61 | 0 | 0.59 | 0.42 |
| Satiety |  |  |  |  |  |
| Hunger | 12 | 7.40 | 0 | −0.43 | 0.10 |
| Satisfaction | 5 | 1.46 | 0 | 0.90 | 0.38 |
| Fullness | 11 | 8.35 | 0 | −0.02 | 0.94 |
| Desire to Eat | 10 | 3.49 | 0 | −0.54 | 0.023 (*) |
| **Females** |  |  |  |  |  |
| Satiation |  |  |  |  |  |
| Hunger | 10 | 2.22 | 0 | 0.13 | 0.24 |
| Satisfaction | 5 | 2.07 | 0 | 0.35 | 0.33 |
| Fullness | 9 | 2.51 | 0 | −0.60 | 0.014 (*) |
| Desire to Eat | 8 | 3.57 | 0 | 0.24 | 0.84 |
| Satiety |  |  |  |  |  |
| Hunger | 10 | 7.43 | 0 | 1.05 | 0.030 (*) |
| Satisfaction | 5 | 2.67 | 0 | 0.38 | 0.65 |
| Fullness | 9 | 10.76 | 0.071 | 0.88 | 0.08 |
| Desire to Eat | 8 | 3.65 | 0 | 0.64 | 0.06 |

(*) = statistically significant, *p*-value < 0.05.

References

1. Olli, K.; Salli, K.; Alhoniemi, E.; Saarinen, M.; Ibarra, A.; Vasankari, T.; Rautonen, N.; Tiihonen, K. Postprandial effects of polydextrose on satiety hormone responses and subjective feelings of appetite in obese participants. *Nutr. J.* **2015**, *14*, 2.
2. Astbury, N.M.; Taylor, M.A.; Macdonald, I.A. Polydextrose results in a dose-dependent reduction in *ad libitum* energy intake at a subsequent test meal. *Br. J. Nutr.* **2013**, *110*, 934–942.
3. Ranawana, V.; Muller, A.; Henry, C.J. Polydextrose: Its impact on short-term food intake and subjective feelings of satiety in males-a randomized controlled cross-over study. *Eur. J. Nutr.* **2013**, *52*, 885–893.
4. Hull, S.; Re, R.; Tiihonen, K.; Viscione, L.; Wickham, M. Consuming polydextrose in a mid-morning snack increases acute satiety measurements and reduces subsequent energy intake at lunch in healthy human subjects. *Appetite* **2012**, *59*, 706–712.
5. Astbury, N.M.; Taylor, M.; Macdonald, I.A. The effects of a polydextrose preload on appetite and energy intake. *Proc. Nutr. Soc.* **2008**, *67*, doi:10.1017/S0029665108000165.
6. Schwab, U.; Louheranta, A.; Torronen, A.; Uusitupa, M. Impact of sugar beet pectin and polydextrose on fasting and postprandial glycemia and fasting concentrations of serum total and lipoprotein lipids in middle-aged subjects with abnormal glucose metabolism. *Eur. J. Clin. Nutr.* **2006**, *60*, 1073–1080.
7. King, N.A.; Craig, S.A.; Pepper, T.; Blundell, J.E. Evaluation of the independent and combined effects of xylitol and polydextrose consumed as a snack on hunger and energy intake over 10 days. *Br. J. Nutr.* **2005**, *93*,
   911–915.
